# Tunable Sound Absorption via Evanescent‐Wave Coupling in Asymmetric Bilayer Metasurfaces

**DOI:** 10.1002/advs.202522261

**Published:** 2026-02-06

**Authors:** Pyung Sik Ma, Hyung Jin Lee

**Affiliations:** ^1^ Department of Mechanical Engineering Gachon University Seongnam Republic of Korea; ^2^ Acoustics, Ultrasound, and Vibration Metrology Group Korea Research Institute of Standards and Science Daejeon Republic of Korea

**Keywords:** acoustic metasurfaces, evanescent waves, near‐field interaction, sound absorption

## Abstract

This study explores tunable sound absorption using a bilayer configuration of phase‐gradient acoustic metasurfaces. By carefully adjusting the cavity length between two metasurface layers, the proposed system can modulate its acoustic response between highly reflective and perfectly absorptive states without changing the internal geometry of the unit cells. The underlying mechanism results from evanescent‐wave coupling, which becomes significant at sub‐wavelength cavity length and is strongly influenced by the phase gradient and integer parity of each metasurface. To analyze the scattering behavior of the bilayer system, an analytical model based on coupled‐mode theory is developed, identifying the conditions that ensure both reflection and transmission are effectively suppressed. Theoretical predictions are validated by full‐wave simulations using bilayer metasurfaces realized with space‐coiling structures. The results demonstrate broadband tunability in sound absorption, with optimal configurations achieving an absorption coefficient exceeding 95%. Owing to its structural simplicity and high tunability, the proposed approach offers an effective solution for dynamic sound control in applications such as tunable noise barriers and reconfigurable sound‐absorbing devices.

## Graphical abstract

Tunable sound absorption is realized through a bilayer metasurface system governed by evanescent‐wave coupling. This work demonstrates that such evanescent‐wave coupling provides a controllable scattering channel that destructively interferes with the specular reflection. By precisely modulating this interaction via interlayer spacing, the system is continuously tuned from a highly reflective state to near‐perfect absorption.

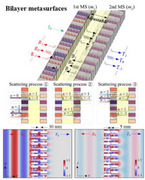

## 1 Introduction

Recent advances in acoustic metasurfaces have enabled unconventional control of sound through phase modulation at sub‐wavelength scales [1, 2, 3]. These engineered surfaces can anomalously redirect transmitted and reflected waves [4, 5, 6], focus acoustic energy within localized regions [7, 8], and steer sound into directional beams [9, 10]. They have also been used to overcome large impedance mismatches between distinct media (e.g., air–water), enabling efficient transmission of acoustic energy across interfaces [11, 12, 13]. In addition, acoustic metasurfaces have demonstrated capabilities such as generating acoustic vortices and converting plane waves into circularly polarized waves [14, 15]. By precisely engineering their local phase profile, acoustic metasurfaces offer wave manipulation functionalities beyond the capabilities of conventional acoustic materials.

In addition to wavefront engineering, acoustic metasurfaces have also been increasingly employed to enhance sound absorption and insulation. Early studies extended phase gradient metasurfaces toward wave attenuation by exploiting the diffraction order and the resulting multiple internal reflections. For example, Qu et al. achieved broadband high absorption by integrating porous materials into metasurface designs [16]. Shen et al. introduced a metasurface design in which multiple internal reflections inside the unit cells enhance sound absorption by increasing the acoustic interaction time [17]. Furthermore, Ju et al. [18] and Song et al. [19] demonstrated asymmetric acoustic transmission and absorption, by utilizing diffraction properties that are strongly dependent on the incidence conditions. Recent studies have increasingly highlighted that higher‐order diffraction and evanescent modes are critical for enhancing metasurface efficiency. Zhu et al. harnessed nonlocal interactions to couple unit cells with distinct resonance frequencies, thereby realizing broadband absorption [20], while Park et al. discussed that these nonlocal interactions are a dominant factor in frequency bands with high absorption [21]. Hou et al. [22] and Li et al. [23] utilized evanescent waves to enhance metasurface capabilities, specifically addressing efficiency degradation at large steering angles and improving retroreflection performance, respectively. Overall, these works demonstrate that advanced wave control is achieved by tailoring phase profiles specifically to manipulate higher‐order diffraction and nonlocal interactions.

Despite the capability of sound control, the acoustic response of most metasurfaces is fixed once their internal structure is designed, limiting their adaptability for dynamic or reconfigurable applications. To overcome this limitation, various approaches have been investigated to introduce tunability into acoustic metasurfaces. For example, Zhang et al. [24] and Gong et al. [25] proposed a motor‐driven resonator within the unit cells, wherein the neck dimensions of the Helmholtz resonator can be adjusted to tune its resonance frequency. Song et al. [26] demonstrated that the effective phase profile within the supercell could be modulated by rotating internal resonators. Ma et al. [27] employed membrane‐type metamaterials with embedded electromagnets to realize adaptive boundary conditions for controlling reverberation characteristics. Li et al. [28] applied a piezoelectric metasurface to achieve dynamic acoustic focusing and reflection control. Zhao et al. [29] proposed a tunable mechanism based on space‐coiling design that enables control over the effective acoustic path length to focus transmitted waves. Zhang et al. [30] proposed an array of substructures with varying properties, in which specific unit cells can be selectively activated to generate holographic acoustic fields. While these adaptive mechanisms can provide high tunability, they often introduce mechanical and electrical complexity and operational costs.

**FIGURE 1:**
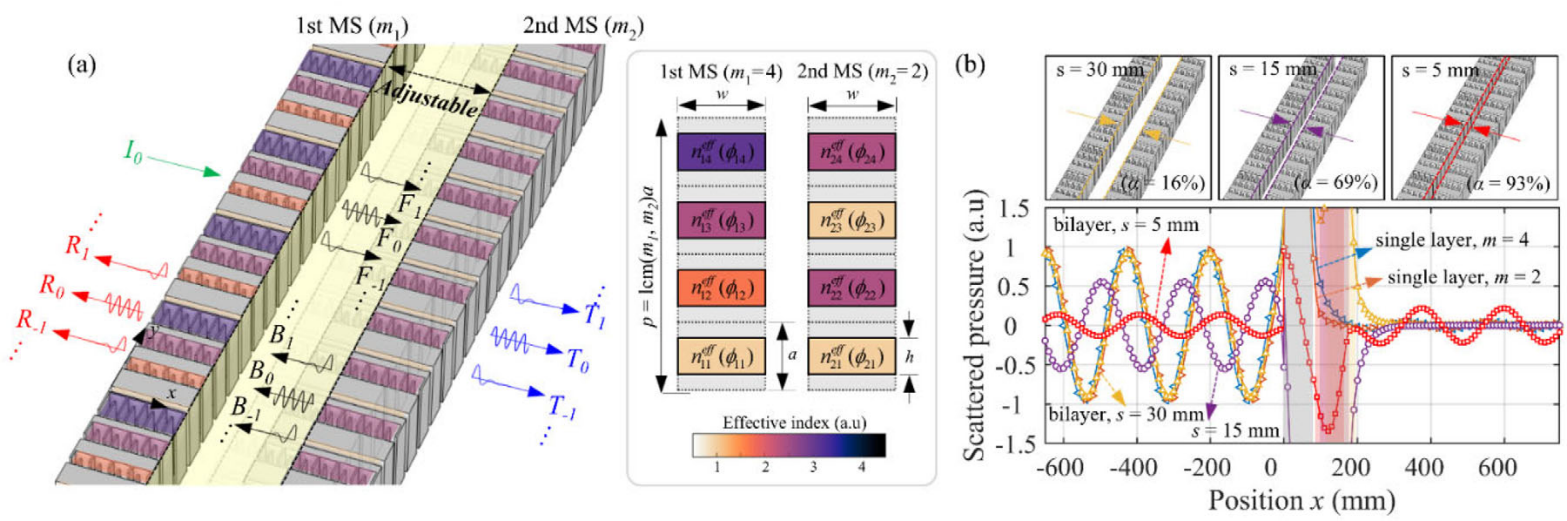
(a) Schematic of the tunable bilayer metasurface configuration. An incident plane wave interacts with two phase‐gradient metasurface layers, separated by a tunable air cavity of length *s*. The interaction generates reflected (*R*_n_) and transmitted (*T*_n_) diffraction orders in the far‐field, and forward (*F*_n_) and backward (*B*_n_) orders within the cavity. The inset shows the effective refractive index profile for an example (*m*_1_, *m*_2_) = (4, 2) configuration. (b) Simulated pressure field for the (4, 2) configuration at 1500 Hz, demonstrating the transition from a highly reflective state (*s* = 30 mm, absorption *α* = 16%) to a perfectly absorptive state (*s* = 5 mm, *α* = 93%) by tuning the cavity length.

To address this challenge, this work proposes a compact bilayer metasurface whose acoustic response is tuned solely by adjusting the sub‐wavelength cavity length between two phase‐gradient layers. Previous studies on bilayer metasurfaces have primarily focused on controlling wave direction or achieving asymmetric transmission, often utilizing large cavity separations where propagating diffraction orders dominate. For instance, Xia et al. [31] realized a broadband acoustic lens using two elastic metasurfaces separated by a wide cavity, and Cao et al. [32] achieved asymmetric transmission of flexural waves with a bilayer metasurface system. Su et al. [33] suggested that bilayer metasurfaces can be used as spatial filters, enabling high‐efficiency transmission of flexural waves only at specific incidence angles. Liu et al. [34] discussed the potential role of evanescent waves between two identical metasurfaces, but the mechanism was limited to asymmetric transmission. In contrast, the present work harnesses the evanescent near‐field, which becomes dominant when two metasurfaces are separated by a sub‐wavelength cavity, as shown in Figure 1a. In this regime, evanescent fields generated by each metasurface can couple across the narrow cavity, fundamentally reshaping the overall scattering behavior of the system. This enables the system to transition from a highly reflective to a strongly absorptive state without modifying any unit‐cell geometry.

The key contribution of this work is the demonstration that evanescent‐wave coupling between two phase‐gradient metasurfaces enables a high‐contrast, continuous tunability of sound absorption over a broad frequency range. Figure 1b presents the simulated scattering for the proposed bilayer system. By varying only the cavity length *s*, the absorption can be tuned continuously from 16% to 93%. At *s* = 30 mm (∼λ0/∼λ07.67.6), most incident energy is reflected and absorption is minimal. The normalized reflection coefficient remains near unity, indicating that the bilayer system behaves as a reflector. As *s* decreases, reflection is reduced due to enhanced near‐field (evanescent) coupling across the cavity, and at an optimal spacing both transmission and reflection are simultaneously suppressed, yielding near‐perfect absorption. Unlike earlier efforts that require a relatively large cavity and provide only binary on/off switching states, the present mechanism achieves continuous control of sound absorption using a single geometric parameter *s*. We systematically examine how the cavity length and metasurface configuration, including phase gradient and integer parity, affect the overall scattering response. To explain the underlying mechanism, we develop an analytical model based on the coupled‐mode theory for bilayer metasurfaces. We then validate the mechanism with full‐wave simulations using a model verified through single‐unit measurements, confirming that the proposed bilayer system offers high absorption tunability while maintaining structural simplicity.

The remainder of this paper is organized as follows: Section 2 describes the configuration of the bilayer metasurface, the coupled‐mode theory formulation, and the absorption mechanism of the bilayer metasurface. The effects of phase gradient and integer parity on the absorption performance of bilayer metasurfaces are also examined. Section 3 investigates broadband absorption, presents the realization of the metasurface units, and verifies tunability through numerical analysis. Section 4 concludes our findings. Section 5 details the experimental methods and numerical techniques used for model validation.

## 2 Sound‐Absorbing Mechanism of Bilayer Metasurfaces

### 2.1 Configuration of Bilayer Metasurfaces

**FIGURE 2:**
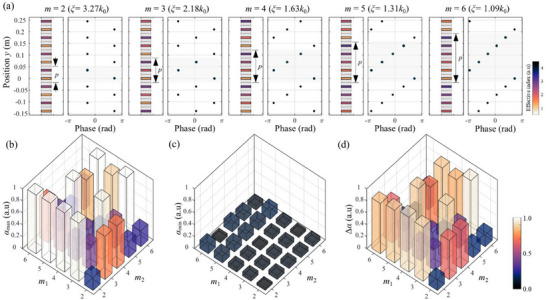
(a) Phase delay profiles and corresponding phase gradients for metasurface layers with *m* varying from 2 to 6. Each metasurface is designed to span a full 2π phase shift across a supercell p at the operating frequency of 1500 Hz. Tunability of sound absorption for bilayer metasurfaces across different (*m*_1_, *m*_2_) combinations at 1500 Hz. (b) Maximum absorption coefficient, *α*_max_. (c) Minimum absorption coefficient, *α*_min_. (d) Tunability range, defined as Δ*α* = *α*_max_ − *α*_min_.

Figure 1a illustrates the configuration of the proposed bilayer structure, which consists of two phase‐gradient metasurfaces placed in parallel and separated by an air cavity. Each metasurface *i* (*i* = 1, 2) comprises *m*_i_ unit cells, and the effective refractive index $n_{\mathrm{ij}}^{\mathrm{eff}}$ of the *j*‐th unit cell is designed such that an acoustic wave passing through metasurface *i* accumulates a total phase shift of 2π at the operating frequency *f*_0_ = 1500 Hz. An example of this design is shown in the inset of Figure 1a, where a bilayer configuration with (*m*_1_, *m*_2_) = (4, 2) is presented. Specifically, the phase shift introduced by the *j*‐th unit cell of metasurface *i* is given by *ϕ*_ij_ = 2π(*j* − 1)/*m*_i_ + *ϕ*_0_, where *ϕ*_0_ is an initial phase offset. This discrete profile results in a linear phase gradient along the *y*‐direction, with a magnitude of *ξ*_*i*_ = *d* *ϕ*/*dy* = 2π/*m*_i_ *a* where *a* denotes the unit‐cell height. In this study, the unit‐cell height *a* is fixed, and the phase gradient is controlled by varying the number of unit cells per period, *m*_i_. The phase‐delay profiles for metasurface layers with *m* varying from 2 to 6, along with the associated phase gradients *ξ*, are summarized in Figure 2a. For the bilayer system, the supercell period of the combined structure is given by *p* = *m*_s_ *a*, where *m*_s_ denotes the least common multiple of *m*_1_ and *m*_2_. Here, we consider normally incident plane waves on the bilayer metasurfaces and assume that the metasurfaces extend infinitely in the *y*‐direction, allowing us to analyze one supercell using periodic boundary conditions. The regions on either side of the bilayer metasurfaces are treated as semi‐infinite with non‐reflecting boundaries to mimic free‐field conditions. Under these assumptions, the acoustic absorption coefficient is defined as *α*   =   1  − *R*  − *T*, where *R* and *T* are the reflectance and transmittance of the bilayer metasurfaces. Note that an ideal absorber would correspond to *α* = 1 (i.e., *R* = *T* = 0), whereas a perfectly reflective system would have *α* = 0 (with *R* = 1, *T* = 0).

When a plane wave is incident on the first metasurface, it generates multiple diffracted waves due to the periodic phase gradient. As illustrated in Figure 1a, the first metasurface reflects part of the wave into various diffraction orders *R*_n_ and transmits forward‐propagating waves *F*_n_ into the cavity. Similarly, the second metasurface generates transmitted wave components *T*_n_ and backward waves *B*_n_ within the cavity. Only the diffraction orders whose transverse wave number $k_{y}^{n}$ is less than the acoustic wave number in air *k*_0_ can propagate into the far field. Higher‐order modes become evanescent and are confined near the metasurface. To enable tunable absorption in the bilayer configuration, we vary only the cavity length *s* between the two metasurface layers. In practical realizations, this single parameter can be controlled by a simple mechanical adjustment, such as using a motor‐driven translation stage or a hydraulic actuation system. Because this tuning relies only on the cavity length *s*, no local actuation or reconfiguration at the unit‐cell level is required. Here, we assume that the separation *s* is uniform across the metasurface aperture. If the cavity length *s* between the metasurfaces is relatively large (comparable to or larger than the wavelength), these evanescent modes decay rapidly and have negligible impact on the scattering characteristics of the bilayer metasurface. However, when the cavity length is sufficiently small (sub‐wavelength), even evanescent higher‐order modes can couple across the cavity and interact significantly with the adjacent metasurface. In such cases, the bilayer system can exhibit strong absorption due to this evanescent‐wave coupling mechanism. With a proper combination of phase gradients and cavity length, it is possible to suppress both reflection and transmission, thereby achieving nearly perfect absorption of the incident acoustic energy. To systematically investigate the sound‐absorbing mechanism of the proposed system, we present the theoretical methods used to analyze these configurations in the following sections.

### 2.2 Coupled‐Mode Theory for Bilayer Metasurfaces

The scattering behavior of a single phase‐gradient metasurface is governed by the generalized Snell's law, where the output channel is determined by the integer parity of the number of unit cells. By the phase gradient of a single metasurface, *ξ*, the tangential wave number increases at each internal travel. When the diffracted wave is above *k*_0_, it becomes evanescent and reflects internally, leading to a stepwise increase in the tangential wave number. The radiating component satisfies the generalized Snell's law summarized as:

(1) $$k_{y}^{\mathrm{sct}}=k_{y}^{\mathrm{inc}}+\left(L-m\right)\xi ,$$

where *L* denotes the number of internal reflections within the metasurface and *m* denotes the number of subunits in a supercell providing a 2π phase span. For normal incidence at 1500 Hz, single metasurface cases for *m* = 2, 3, and 4 give *L* = *m*. Thus, while the scattered wave number $k_{y}^{\mathrm{sct}}$ is identical to the incident component $k_{y}^{\mathrm{inc}}$, the output channel is selected by the parity of *m*—single metasurfaces of *m* = 2, 4 (even) produce an upstream back‐scattered (reflected) output, whereas *m* = 3 (odd) yields a downstream transmitted output. However, the generalized Snell's law for the single metasurface does not capture the complex interactions in a bilayer system where near‐field coupling is significant.

To quantitatively predict the scattering characteristics of the bilayer metasurfaces, a coupled‐mode theory (CMT) formulation [3, 35, 36] is employed to model the interaction between metasurfaces. The system is divided into three regions: Region I (upstream), Region II (air cavity between the two metasurfaces), and Region III (downstream). Due to the periodicity in the *y*‐direction, the acoustic fields in these regions are expanded into plane wave components with reciprocal wave number αn=ky+2πn/2πnpp(*n* = −∞,  ...,  ∞), including both propagating and evanescent modes. Assuming a time‐harmonic term *e* ^jωt^ with angular frequency *ω* = 2π*f_0_*, the total pressure fields in each region are represented as:

(2) $$\begin{array}{ccc} & & p_{up}\left(x,y\right)=I_{0}e^{-j(k_{x}x+k_{y}y)}+\sum_{n=-\infty }^{\infty }R_{n}e^{jk_{x}^{n}x}e^{-j\alpha_{n}y}\\ & & p_{cav}\left(x,y\right)=\sum_{n=-\infty }^{\infty }\left[F_{n}e^{-jk_{x}^{n}\left(x-w\right)}+B_{n}e^{jk_{x}^{n}\left(x-w-s\right)}\right]e^{-j\alpha_{n}y}\\ & & \text{and}\;p_{down}\left(x,y\right)=\sum_{n=-\infty }^{\infty }T_{n}e^{-jk_{x}^{n}\left(x-2w-s\right)}e^{-j\alpha_{n}y} \end{array}$$

where *k*_x_ = *k*_0_cos θ_i_, *k*_y_ = *k*_0_sin θ_i_, *k*_0_ = ω/*c*_0_, $\alpha_{n}=k_{0}\sin\theta_{i}+2\pi n/p,k_{x}^{n}=\sqrt{{k_{0}}^{2}-{\alpha_{n}}^{2}}$, and θ_i_ is the angle of incidence. Due to the subwavelength dimensions of the metasurface unit cells (*w* ≈ λ_0_/28.6), we assume that only the fundamental waveguide mode propagates within each unit cell. As illustrated in the inset of Figure 1a, each unit cell of the metasurface has an effective refractive index $n_{\mathrm{ij}}^{\mathrm{eff}}$, which yields the local wave number $k_{\mathrm{ij}}=n_{\mathrm{ij}}^{\mathrm{eff}}\cdot k_{\mathrm{sc}}$. Thus, the internal pressure field for the *j*‐th unit cell of each metasurface *i* is expressed as

(3) $$\begin{array}{ccc} p_{MS1,j}\left(x\right) & = & a_{j}e^{-jk_{1j}x}+b_{j}e^{jk_{1j}\left(x-w\right)},\;p_{MS2,j}\left(x\right)=c_{j}e^{-jk_{2j}\left(x-w-s\right)}\\ & & +d_{j}e^{jk_{2j}\left(x-2w-s\right)} \end{array}$$

Here, *k*_sc_ denotes the nominal wave number within the channel of height *h*. To account for viscous and thermal dissipation in narrow channels of space‐coiling structures, the effective density ρ_sc_ and compressibility *C*_sc_ were calculated by [37], and the wave number was calculated as $k_{\mathrm{sc}}=\omega \sqrt{\rho_{\mathrm{sc}}C_{\mathrm{sc}}}$.

Accordingly, the velocity field in the *x*‐direction can be derived from Euler's equation for upstream, cavity, downstream, and unit cells as:

(4) $$\begin{array}{ccc} & & v_{x,up}\left(x,y\right)=\frac{1}{\omega \rho_{0}}\left(k_{x}I_{0}e^{-j(k_{x}x+k_{y}y)}-\sum_{n=-\infty }^{\infty }k_{x}^{n}R_{n}e^{jk_{x}^{n}x}e^{-j\alpha_{n}y}\right)\\ & & v_{x,cav}\left(x,y\right)=\frac{1}{\omega \rho_{0}}\sum_{n=-\infty }^{\infty }k_{x}^{n}\left[F_{n}e^{-jk_{x}^{n}\left(x-w\right)}-B_{n}e^{jk_{x}^{n}\left(x-w-s\right)}\right]e^{-j\alpha_{n}y}\\ & & \text{and}\;v_{x,down}\left(x,y\right)=\frac{1}{\omega \rho_{0}}\sum_{n=-\infty }^{\infty }k_{x}^{n}T_{n}e^{-jk_{x}^{n}\left(x-2w-s\right)}e^{-j\alpha_{n}y} \end{array}$$

(5) $$\begin{array}{ccc} & & v_{x,MS1}\left(x\right)=\left\{\begin{array}{c} \frac{k_{1j}}{\omega \rho_{1j}}\left(a_{j}e^{-jk_{1j}x}-b_{j}e^{jk_{1j}\left(x-w\right)}\right)\;\;\left(\left|y-y_{j}\right|\leq h/2\right)\\ 0\;\;\;\;\;\left(h/2\leq \left|y-y_{j}\right|\leq a/2\right) \end{array}\right.\\ & & \text{and}\\ & & v_{x,MS2}\left(x\right)\\ & & \;=\left\{\begin{array}{c} \frac{k_{2j}}{\omega \rho_{2j}}\left(c_{j}e^{-jk_{2j}\left(x-w-s\right)}-d_{j}e^{jk_{2j}\left(x-2w-s\right)}\right)\;\;\left(\left|y-y_{j}\right|\leq h/2\right)\\ 0\;\;\;\;\;\;\left(h/2\leq \left|y-y_{j}\right|\leq a/2\right) \end{array}\right. \end{array}$$

where *ρ*_0_ denotes the density of air, *y*_j_ (= (*j* − 1)*a*) is the center position in the *y*‐direction, and *ρ*_ij_ is equivalent density of *j*‐th unit cell of metasurface *i*. At the boundary between upstream and the first metasurface (*x* = 0), continuity conditions for pressure and *x*‐component of the velocity are satisfied as:

(6) $$\begin{array}{ccc} & & I_{0}e^{-jk_{y}y}+\sum_{n=-\infty }^{\infty }R_{n}e^{-j\alpha_{n}y}=a_{j}+b_{j}e^{-jk_{1j}w}\\ & & \times \left(\left|y-y_{j}\right|\leq h/2,j=1,2,\text{…},m_{s}\right)\\ & & \text{and}\\ & & \frac{1}{\rho_{0}}\left(k_{x}p_{0}e^{-jk_{y}y}-\sum_{n=-\infty }^{\infty }k_{x}^{n}R_{n}e^{-j\alpha_{y}y}\right)\\ & & \;=\left\{\begin{array}{c} \frac{k_{1j}}{\rho_{1j}}\left(a_{j}-b_{j}e^{-jk_{1j}w}\right)\;\;\;\;\;\left(\left|y-y_{j}\right|\leq h/2\right)\\ 0\;\;\;\;\;\;\;\;\;\;\left(h/2\leq \left|y-y_{j}\right|\leq a/2\right) \end{array}\right. \end{array}$$

Integrating Equation 6 over the supercell of metasurface in the *y*‐direction and using the orthogonality between plane waves, gives the relations for the reflected waves and unit cell amplitudes *R*_n_, *a*_j_, and *b*_j_

(7) $$\begin{array}{ccc} & & \sum_{n=-\infty }^{\infty }\left(\delta_{n,0}+R_{n}\right)e^{-j\alpha_{n}y_{j}}\mathrm{sinc}\left(\frac{\alpha_{n}d}{2}\right)=a_{j}+b_{j}e^{-jk_{1j}w}\;\;\left(j=1,2,\text{…},m_{s}\right)\\ & & \frac{1}{\rho_{0}}k_{x}^{n}\left(\delta_{n,0}-R_{n}\right)=\sum_{j=1}^{m_{s}}\frac{1}{\rho_{1j}}k_{1j}\left(a_{j}-b_{j}e^{-jk_{1j}w}\right)e^{j\alpha_{n}y_{j}}\frac{h}{a}\mathrm{sinc}\left(\frac{\alpha_{n}h}{2}\right) \end{array}$$

The same procedure is applied to the interface between the first metasurface and the cavity at *x* = *w*, resulting in

(8) $$\begin{array}{ccc} & & \sum_{n=-\infty }^{\infty }\left(F_{n}+B_{n}e^{-jk_{x}^{n}s}\right)e^{-j\alpha_{n}y_{j}}\mathrm{sinc}\left(\frac{\alpha_{n}h}{2}\right)\\ & & \;=a_{j}e^{-jk_{1j}w}+b_{j}\;\;\left(j=1,2,\text{…},m_{s}\right)\\ & & \text{and}\\ & & \frac{1}{\rho_{0}}k_{x}^{n}\left(F_{n}-B_{n}e^{-jk_{x}^{n}s}\right)\\ & & \;=\sum_{j=1}^{m_{s}}\frac{1}{\rho_{1j}}k_{1j}\left(a_{j}e^{-jk_{1j}w}-b_{j}\right)e^{j\alpha_{n}y_{j}}\frac{h}{a}\mathrm{sinc}\left(\frac{\alpha_{n}h}{2}\right) \end{array}$$

Similarly, the continuity conditions at the second metasurface boundaries, both on the cavity (*x* = *w* + *s*) and the downstream side (*x* = 2*w* + *s*) are satisfied respectively as:

(9) $$\begin{array}{ccc} & & \sum_{n=-\infty }^{\infty }\left(F_{n}e^{-jk_{x}^{n}s}+B_{n}\right)e^{-j\alpha_{n}y_{j}}\mathrm{sinc}\left(\frac{\alpha_{n}h}{2}\right)\\ & & \;=c_{j}+d_{j}e^{-jk_{2j}w}\;\left(j=1,2,\text{…},m_{s}\right)\\ & & \text{and}\\ & & \frac{1}{\rho_{0}}k_{x}^{n}\left(F_{n}e^{-jk_{x}^{n}s}-B_{n}\right)\\ & & \;=\sum_{j=1}^{m_{s}}\frac{1}{\rho_{2j}}k_{2j}\left(c_{j}-d_{j}e^{-jk_{2j}w}\right)e^{j\alpha_{n}y_{j}}\frac{h}{a}\mathrm{sinc}\left(\frac{\alpha_{n}h}{2}\right) \end{array}$$

(10) $$\begin{array}{ccc} & & \sum_{n=-\infty }^{\infty }T_{n}e^{-j\alpha_{n}y_{j}}\mathrm{sinc}\left(\frac{\alpha_{n}h}{2}\right)\\ & & \;=c_{j}e^{-jk_{2j}w}+d_{j}\;\left(j=1,2,\text{…},m_{s}\right)\\ & & \text{and}\\ & & \frac{1}{\rho_{0}}k_{x}^{n}T_{n}\\ & & \;=\sum_{j=1}^{m_{s}}\frac{1}{\rho_{2j}}k_{2j}\left(c_{j}e^{-jk_{2j}w}-d_{j}\right)e^{j\alpha_{n}y_{j}}\frac{h}{a}\mathrm{sinc}\left(\frac{\alpha_{n}h}{2}\right) \end{array}$$

For numerical implementation, the infinite set of modes is truncated to a finite number of diffraction orders 2*n*_t_ + 1. Solving Equations (7), (8), (9), (10) provides the amplitudes of diffraction orders *R*_n_, *F*_n_, *B*_n_, and *T*_n_ (for *n* = −*n*_t_,…,   0, …,   *n*_t_) and unit cell amplitudes *a*_j_, *b*_j_, *c*_j_, *d*_j_ (for *j* = 1, …,   *m*_s_) for each frequency. The acoustic absorption performance of the bilayer metasurface *α* is finally evaluated by computing the total acoustic energies associated with reflected and transmitted waves as:

(11) $$R=-\sum_{n=-n_{t}}^{n_{t}}\alpha_{n}R_{n}R_{n}^{*}/2\omega \rho_{0}\;\mathrm{and}\;T=-\sum_{n=-n_{t}}^{n_{t}}\alpha_{n}T_{n}T_{n}^{*}/2\omega \rho_{0}$$

where ^*^ represents the complex conjugate.

### 2.3 Sound‐Absorbing Mechanism via Evanescent‐Wave Coupling

**FIGURE 3:**
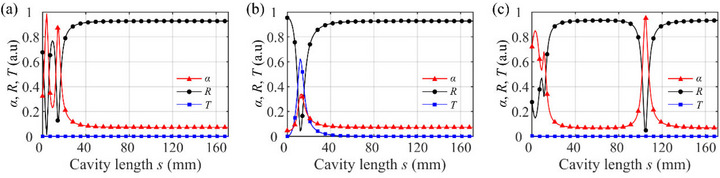
Absorption (triangles), reflectance (circles), and transmittance (squares) as a function of cavity length *s* at 1500 Hz for three bilayer configurations: (a) (*m*_1_, *m*_2_) = (4, 2), (b) (4, 4), and (c) (3, 2).

To systematically evaluate how phase gradients affect tunable absorption, the CMT analysis was applied to all combinations of *m*_1_ and *m*_2_ ranging from 2 to 6 at the operating frequency of 1500 Hz. For each configuration, the cavity length *s* was varied from 0 to 0.75λ_0_, and the corresponding absorption coefficients were calculated. Figure 2 summarizes the maximum and minimum absorption coefficients and the resulting tunability (Δ*α* = *α*_max_ − *α*_min_. The results clearly show that several asymmetric configurations, such as (3, 2), (3, 4), (3, 6), (4, 2), (5, 2), (5, 4), and (5, 6), exhibit high tunability exceeding 85%, allowing the system to transition from a highly reflective to a perfectly absorptive state. See Section S.1 for order‐resolved amplitudes of scattered waves for each combination. The dependence on asymmetry is illustrated with three representative cases in Figure 3. For an asymmetric even‐even configuration (*m*_1_, *m*_2_) = (4, 2), the absorption coefficient changes dramatically with cavity length as shown in Figure 3a. At *s* = 4 mm, the absorption coefficient exceeds 95%, indicating that nearly all incident sound energy is absorbed. In contrast, for the symmetric configuration (*m*_1_, *m*_2_) = (4, 4), the absorption is much less sensitive to the cavity length, reaching a maximum of only 34% (Figure 3b). This suggests that a mismatch in phase gradients between the two layers is essential to achieving perfect absorption. Finally, for the asymmetric odd–even case (*m*_1_, *m*_2_) = (3, 2), near‐perfect absorption is also achievable, but the optimal cavity length occurs at a much larger separation. The absorption mechanism is critically dependent on whether propagating or evanescent diffracted waves dominate the interaction within the cavity. This distinction explains the different behaviors observed for even–even and odd–even configurations. In the following subsections, we examine the underlying principles of the absorption characteristics for different metasurface combinations, categorized by their respective mechanisms.

#### 2.3.1 Asymmetric Even–Even Configurations

We begin with a (*m*_1_, *m*_2_) = (4, 2) configuration (both *m*_1_, *m*_2_ even but *m*_1_ ≠ *m*_2_) that exhibits large tunability. As shown in Figure 3a, the bilayer metasurface with (*m*_1_, *m*_2_) = (4, 2) exhibits near‐perfect absorption at *s* = 4 mm that is only about 2% of the wavelength (*λ*_0_ = 229 mm at 1500 Hz), yet it produces a considerable change in absorption performance. After another absorption peak at *s* = 14 mm, the absorption gradually declines as *s* increases; once *s* exceeds approximately 50 mm, the absorption levels off at a low value (∼7.5%), with most of the acoustic energy being reflected. Note that each metasurface individually would reflect nearly all the incident energy (only a small portion is dissipated internally), yielding absorptions of 7.2% and 6.9% for *m* = 4 and 2, respectively. This example demonstrates that by simply adjusting the sub‐wavelength cavity between two asymmetric metasurfaces, one can continuously control the acoustic performance of the system from a nearly perfect absorption state to a highly reflective state.

**FIGURE 4:**
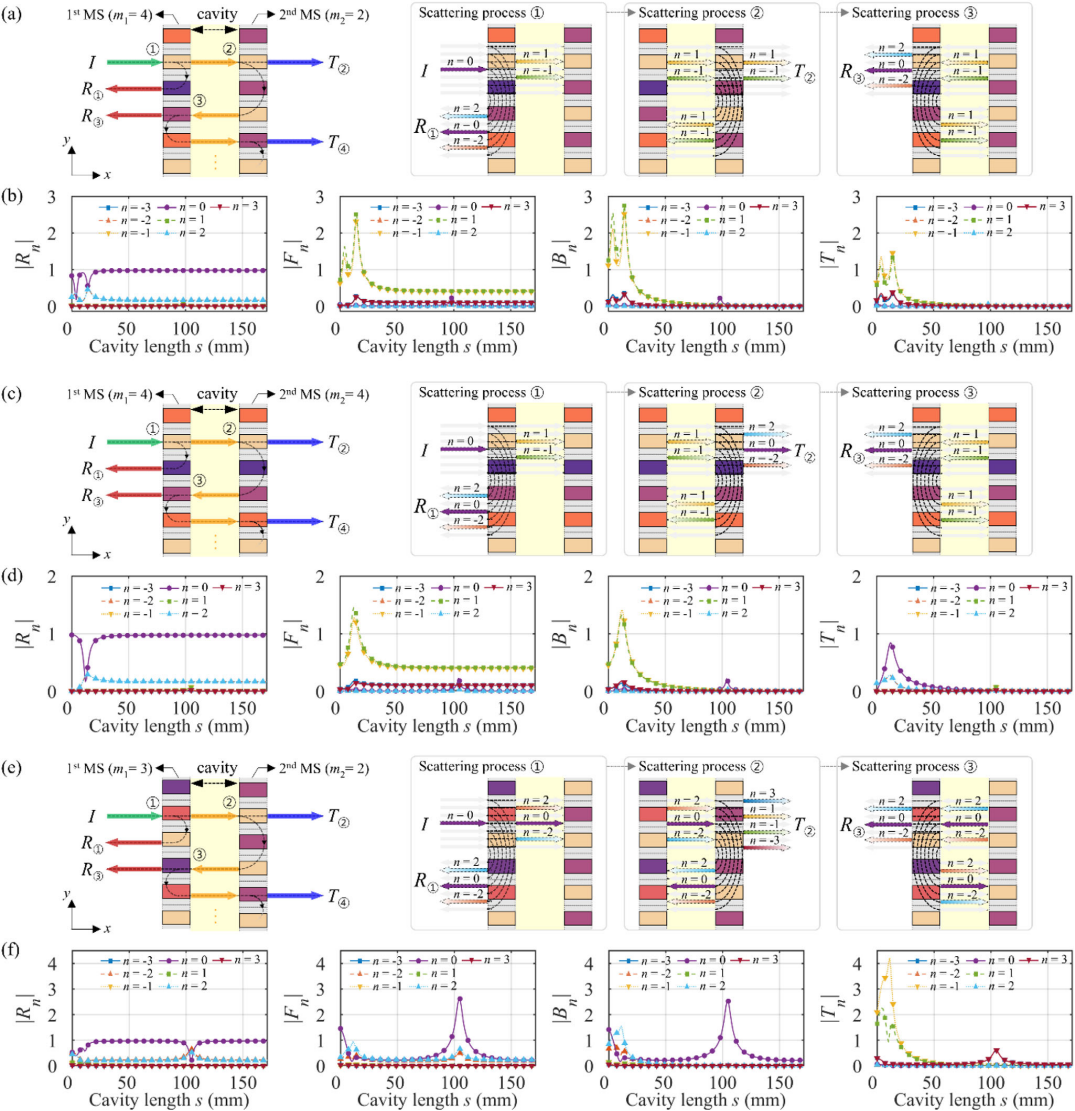
Detailed analysis of the sound absorption mechanism. Schematic illustrations of the successive scattering events (processes ①, ②, ③) for (a) (*m*_1_, *m*_2_) = (4, 2), (c) (4, 4), and (e) (3, 2). The insets highlight the dominant evanescent modes and how they are scattered at each metasurface layer. (b), (d), (f) Amplitudes of the reflected (∣*R*_n_∣), transmitted (∣*T*_n_∣), forward (∣*F*_n_∣), and backward (∣*B*_n_∣) wave components as a function of the cavity length *s* at 1500 Hz for the corresponding configurations. The results confirm the suppression of the 0th‐order far‐field components (*R*_0_ and *T*_0_) at the optimal *s*.

To understand evanescent‐wave coupling, it is helpful to examine the scattering behavior of each metasurface individually. Figure 4a schematically depicts the successive scattering events for normal incidence: scattering at the first metasurface (process ①), at the second metasurface (process ②), and the round trip back to the first metasurface (process ③). For clarity, the insets decompose the reflected and transmitted fields at each step into diffraction orders (index *n*). Note that except for the fundamental *n* = 0 component, all orders are evanescent because their tangential wave number $|\alpha_{n}|>k_{0}$. First, we examine a single metasurface using the CMT model by evaluating the order‐resolved scattering of each metasurface with *m* = 4 and *m* = 2, and highlight the dominant components in the insets of Figure 4a. To verify this evanescent coupling behavior, we numerically excite a single metasurface with higher‐order incident wave components and analyze the resulting scattering characteristics [38]. The single‐layer responses used for the insets are summarized in Section S.2.

As shown in the scattering process ① of Figure 4a, an *m* = 4 metasurface reflects an incident wave into the fundamental 0th‐order wave (purple arrow in *R*_①_). The ±1 diffraction orders transmit through this process, but the transverse wave number exceeds the free‐space wave number, so they are evanescent. When the ±1‐order evanescent waves are incident upon the *m* = 2 metasurface, the ±1‐order evanescent waves are either reflected back or transmitted as evanescent waves (see the process ②), so downstream transmission is effectively suppressed. The ±1‐order evanescent waves reflected by second metasurface (*m*_2_ = 2) then return to the first metasurface (process ③), where they are scattered again and can contribute to a 0th‐order propagating wave. The 0th‐order wave (*R*_①_) directly reflected from the first metasurface (*m*_1_ = 4) acts as a background field that is present across a wide frequency band. In parallel, the resonance arising from evanescent waves between the two metasurfaces also generates a 0th‐order reflected wave (*R*_③_). High absorption is activated when these 0th‐order reflected waves destructively interfere. Therefore, nearly perfect absorption can be achieved by simultaneously suppressing both the reflected and transmitted waves at an optimal value of *s*. To further validate the proposed mechanism, we examine the detailed distribution of diffraction order amplitudes for the bilayer metasurfaces. Figure 4b presents the magnitude of each diffracted component for the (*m*_1_, *m*_2_) = (4, 2) bilayer as a function of cavity length *s*. Consistent with the above explanation estimated from the single process, both the 0th‐order reflection (*R*_0_) and transmission (*T*_0_) components are completely suppressed at the optimal *s* = 4 and 14 mm. In addition, the ±1 evanescent orders are clearly present within the cavity (*F*_±1_ and *B*_±1_) and in the downstream region (*T*_±1_), and their amplitudes peak at these optimal *s*’s.

When the order of the (4, 2) configuration is reversed to (2, 4) (equivalent to the incidence direction changing from downstream to upstream), it can be confirmed that the absorption tunability is not significant. As shown in Section S.5, the incident wave on the 1st metasurface (*m*_1_ = 2) is reflected as the 0th diffraction order component and transmitted into the cavity as ±2nd order components. However, these evanescent components are then scattered back by the second metasurface (*m*_2_ = 4) into 0th and ±2nd ‐order components. Therefore, when the cavity is at sub‐wavelength, the cavity length is insufficient to induce destructive interference for high absorption. Furthermore, for the case of the half‐wavelength cavity, the involved ±2 order components all decay, making it impossible to obtain sufficient absorption.

#### 2.3.2 Symmetric Configurations

In contrast, for the (*m*_1_, *m*_2_) = (4, 4) configuration, the ±1‐order evanescent waves diffracted by the first metasurface (*m*_1_ = 4) can be converted into a propagating 0th‐order wave by the second metasurface (*m*_2_ = 4), as shown in the scattering process ② of Figure 4c. As shown in Figure 4d, it is found that a 0th‐order wave can indeed propagate downstream due to the near‐field coupling associated with *n* = ±1 evanescent waves. Since both layers in the (4, 4) case have identical scattering characteristics, evanescent fields produced by the first layer will be converted back into a 0th‐order wave by the 2nd layer due to reciprocity. As a result, some acoustic energy inevitably leaks through as a transmitted wave. Consequently, the maximum absorption for the (4, 4) bilayer metasurfaces remains below 50%, even at the optimal cavity length. See the Section S.1.1 for amplitudes of scattered waves for symmetric combinations, showing the 0th‐order wave in the transmission coefficient. For symmetric even–even configurations, the 0th‐order transmission appears due to the evanescent‐wave coupling between two symmetric metasurfaces at the sub‐wavelength *s*. For symmetric odd–odd configurations, the transmission peak of 0th‐order wave is apparent at the half wavelength *s* as the single metasurface can produce the downstream transmission wave.

#### 2.3.3 Asymmetric Odd–Even Configurations

For the bilayer metasurface (*m*_1_, *m*_2_) = (3, 2), where *m*_1_ is odd, *m*_2_ is even, as shown in Figure 3c, the bilayer configuration exhibits high absorption, but the optimal cavity length occurs at about half a wavelength (*s* = 105 mm). As shown in Figure 4e,f, both the 0th‐order reflected and transmitted components are suppressed when the cavity length *s* = 105 mm, resulting in high absorption performance. At the sub‐wavelength cavity length, the absorption coefficient for (3, 2) peaks around 85% (lower than the perfect absorption achieved in the (4, 2) case). Unlike the earlier configurations with *m*_1_ = 2 or 4 (even), the *m*_1_ = 3 metasurface allows 0th‐order transmission, as illustrated in the scattering process ① of Figure 4e. As a result, in the (3, 2) bilayer metasurface, the 0th‐order components appear within the cavity (*F*_0_ and *B*_0_ in Figure 4f). Consequently, a half‐wavelength cavity length is needed to achieve destructive interference of the 0th‐order reflections. This scenario, in which the 0th‐order mode transmitted into the cavity is reflected by the second metasurface at a separation corresponding to the half‐wavelength condition, is consistent with the well‐known Fabry–Perot resonance [39]. Minor absorption peaks associated with ±2 evanescent orders are also observed at smaller cavity lengths. However, the absorption at these peaks is slightly lower than the main peak, due to the simultaneous excitation of fundamental and higher‐order modes. See the Section S.1.3 for amplitudes of scattered waves for odd–even combinations, showing the consistent absorption peak near the half wavelength *s*.

Overall, the absorption performance of bilayer metasurfaces is governed by the interplay between the cavity length and the integer parity of each layer (and thus the resulting phase gradient). Although the unit‐cell height *a* is fixed in this section, the effect of varying *a* on the absorption performance is systematically investigated in Section S.6. To utilize these factors for high absorption, it is crucial to understand the scattering characteristics of each layer and to properly engineer the inter‐layer interactions involving multiple diffraction orders. Specifically, high absorption is achieved when the suppression of the 0th‐order propagating mode is accompanied by the excitation of higher‐order evanescent components, which effectively confines the incident energy in the near‐field. This underlying mechanism is robust beyond specific gradient designs, as further demonstrated by Section S.7, where the metasurfaces with arbitrary phase distributions exhibit consistent absorption mechanisms. However, a distinct feature of the proposed bilayer system is its capability to achieve continuous tunability without structural modification. Unlike reconfigurable metasurfaces relying on phase‐delay tuning, our approach harnesses the evanescent‐wave coupling as a control parameter to manipulate the acoustic response solely by adjusting the cavity spacing *s*.

## 3 Results and Discussion

### 3.1 Broadband Absorption Performance

**FIGURE 5:**
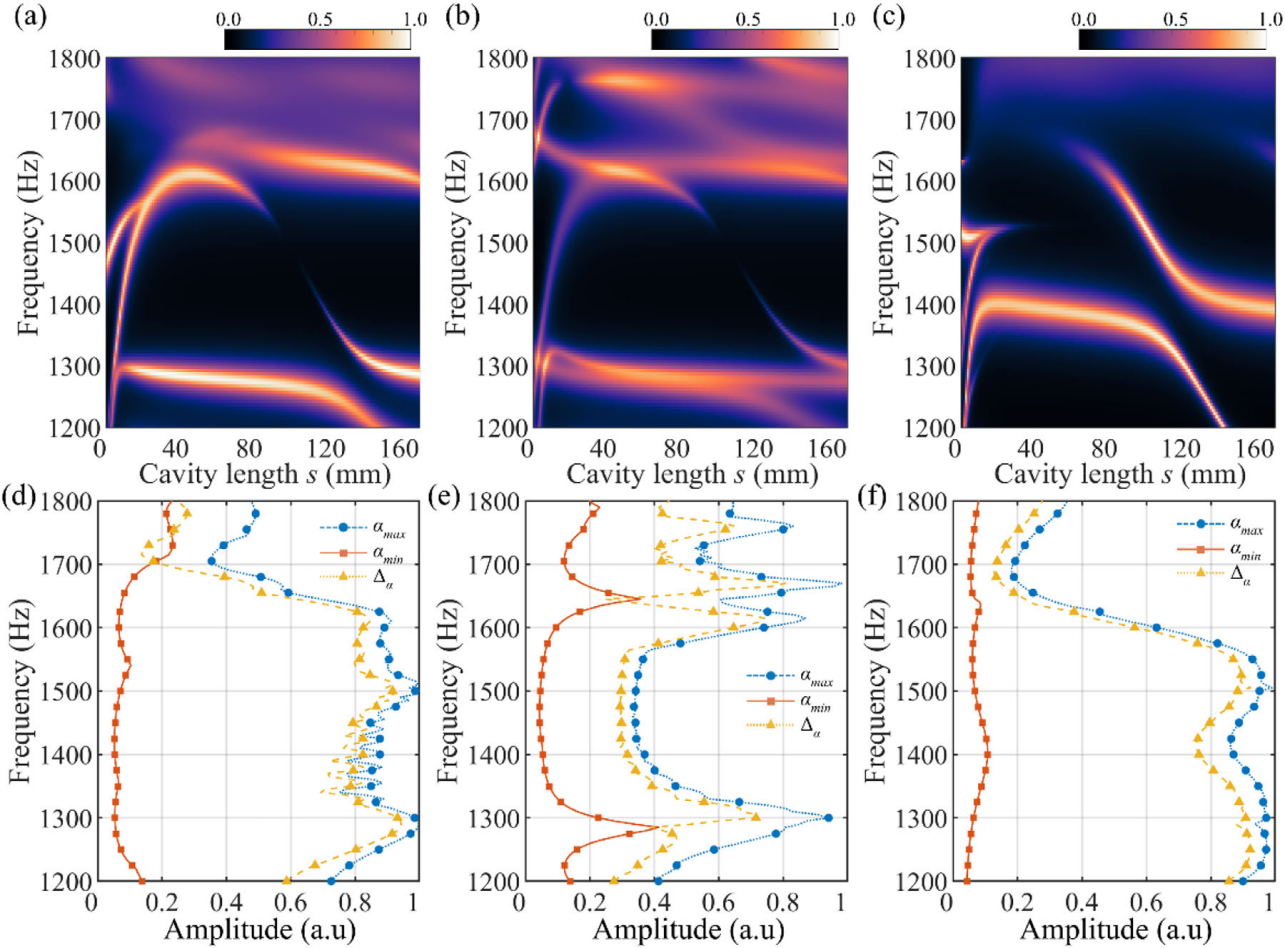
(a–c) Sound absorption performance over a broad frequency range for three bilayer configurations: (*m*_1_, *m*_2_) = (a) (4, 2), (b) (4, 4), and (c) (3, 2). The vertical axis is frequency and the horizontal axis is cavity length *s*. (d–f) Tunability of sound absorption over a broad frequency range for bilayer metasurface configurations of (*m*_1_, *m*_2_) = (d) (4, 2), (e) (4, 4), and (f) (3, 2). Each plot shows the maximum, minimum absorption coefficient as a function of frequency, and the resulting tunability Δ*α* = *α*_max_ − *α*_min_.

Although the metasurfaces were specifically designed for an operating frequency *f*_0_ of 1500 Hz, we investigate whether the proposed bilayer configurations can maintain high absorption performance over a broader frequency range. Using the CMT model, we analyzed the frequency dependence of absorption performance over the frequency range 1–2 kHz for several representative configurations while also varying the cavity length *s*. Figure 5a presents a colormap of the absorption coefficient for (*m*_1_, *m*_2_) = (4, 2) configuration as a function of frequency (vertical axis) and cavity length *s* (horizontal axis). At the operating frequency of 1500 Hz, a high absorption region appears at sub‐wavelength cavity lengths of 4 and 14 mm. As the frequency decreases, the optimal cavity length for peak absorption gradually shifts to smaller values for effective near‐field coupling. When *s* is less than 20 mm, two distinct absorption branches are observed due to strong near‐field coupling between the two metasurfaces. To identify the contribution of individual unit cells to each absorption branch, we examined the amplitudes of the unit cells (*a*_j_, *b*_j_, *c*_j_, and *d*_j_ for *j* = 1 to *m*_s_) within the metasurfaces. The detailed variation of these amplitudes as a function of frequency and cavity length *s* is presented in Figure S28 of Section S.3. From the analysis, it is observed that for a given cavity length, the lower‐frequency branch is primarily excited by the amplitudes of the 1st and 3rd unit cells of the first metasurface and the 1st unit cell of the second metasurface. The higher‐frequency branch, in contrast, is found to be a result of the coupling between the 2nd and 4th unit cells of the first metasurface and the 2nd unit cell of the second metasurface. This indicates that the two absorption branches correspond to distinct resonance channels, formed by the hybridization of specific subunits across the two metasurfaces. As *s* increases beyond this regime, the branches gradually merge into a single absorption peak.

Importantly, the (4, 2) configuration maintains strong absorption over a broad frequency band. As shown in Figure 5d, the tunability for absorption performance remains above 80% across the frequency range from 1250 to 1625 Hz. This broad bandwidth is particularly notable given that the metasurfaces were designed for a single frequency, indicating the robustness of the evanescent coupling mechanism. Furthermore, the difference between maximum and minimum absorption at each frequency (i.e., the absorption tunability) remains substantial throughout most of this band. Figure 5b shows the absorption map for the (4, 4) configuration. As expected, since this configuration does not fully suppress the 0th‐order wave, its overall absorption is lower than that of the (4, 2) case. Figure 5e confirms that the tunability remains below 80% across all frequencies. Moreover, the dependence on cavity length is relatively weak, as presented in Figure 5b, showing that changes in the cavity length do not significantly affect the absorption, except near certain resonance conditions. Even when a local resonance or standing wave occurs at a specific frequency, the absorption of the (4, 4) configuration is not substantially affected by changing *s*, resulting in limited tunability over the broadband frequency range. In contrast, Figure 5c shows that the (*m*_1_, *m*_2_) = (3, 2) supports broad absorption bands at cavity lengths around half‐wavelength. These peaks are attributed to destructive interference of the 0th‐order mode, as discussed earlier. When *s* is around half wavelength, the absorption exceeds 95%, and the bilayer system maintains absorption tunability above 80% from 1090 to 1570 Hz (Figure 5f). Meanwhile, near the operating frequency (1500 Hz), evanescent coupling at sub‐wavelength *s* still enables significant absorption—such as the 85% observed at *s* = 3 mm. However, beyond this frequency range, evanescent‐wave coupling contributes less, and the absorption behavior is increasingly governed by 0th‐order mode interference.

Table 1 summarizes the broadband absorption characteristics for bilayer metasurface combinations. For *m*_1_ and *m*_2_ ranging from 2 to 6, the frequency bandwidths over which the absorption tunability (Δ*α* = *α*_max_ − *α*_min_) exceeds 80% were evaluated. It quantitatively confirms that asymmetric even‐even configurations like (4, 2) and (6, 2) achieve high tunability within the sub‐wavelength, near‐field regime (values in parentheses). In contrast, for odd‐even configurations like (3, 2) and (5, 2) combinations require half‐wavelength cavities to achieve broad tunability via Fabry–Perot resonance.

**TABLE 1:** Frequency bandwidth of high absorption tunability (> 80%) for bilayer metasurfaces categorized by (*m*_1_, *m*_2_) parity combinations. The bandwidth is normalized by the operating frequency *f*_0_ = 1500 Hz. Values in parentheses indicate the bandwidth when the cavity length *s* is resctricted to the near‐field regime (*s* < *λ*_0_/5), highlighting configurations where absorption tunability is dominated by evanescent‐wave coupling.

*m*_2/_ *m*_1_	2	3	4	5	6
2	0.0% (0.0%)	2.7%(2.7%)	2.0%(2.0%)	0.0%(0.0%)	0.3%(0.0%)
3	19.7%(1.7%)	0.0%(0.0%)	9.3%(0.0%)	0.0%(0.0%)	13.0%(3.0%)
4	20.0%(14.3%)	1.7%(0.7%)	0.3%(0.3%)	1.0%(0.3%)	2.7%(1.3%)
5	13.3%(0.7%)	0.0%(0.0%)	10.0%(0.0%)	3.3%(1.0%)	6.7%(0.0%)
6	13.7%(10.0%)	5.3%(0.7%)	0.3%(0.0%)	0.0%(0.0%)	0.0%(0.0%)

### 3.2 Asymmetric Absorption

In Section 2.3, we examined how the absorption of bilayer metasurfaces depends on the layer configuration and identified the underlying mechanism based on the scattering and coupling of higher‐order diffracted modes, including evanescent components. As shown in Figure 2d , the absorption map is not symmetric with respect to the *m*_1_ = *m*_2_ line, indicating that a bilayer with (*m*_1_, *m*_2_) does not generally exhibit the same response as its reversed counterpart (*m*_2_, *m*_1_). These two configurations are mirror images of each other and can be regarded as the same bilayer system excited by opposite incidence. Because each metasurface layer is passive and the overall system is reciprocal, the transmission for opposite incidence directions remains identical, whereas the reflection can differ, leading to asymmetric absorption.

Importantly, such asymmetric behavior is not limited to odd–even combinations. Even for an even–even pair such as (*m*_1_, *m*_2_) = (4, 2), the absorption (and reflection) changes when the incidence direction is reversed (equivalently, when the layer order is reversed to (2, 4)). To clarify the origin of this asymmetry, we compare the internal diffracted fields. For (*m*_1_, *m*_2_) = (4, 2), our analysis in Figure 4a showed that multiple scattering between the two layers results in a cavity field dominated by the 𝑛 = ±1 components. In contrast, for the reversed configuration (*m*_1_, *m*_2_) = (2, 4) (equivalently, backward incidence on (4, 2)), the first layer primarily generates the 𝑛 = ±2 diffracted components, which are then scattered by the second layer to produce the *n* = 0 component. Although the propagating components observed outside the bilayer may appear similar, the distinct evanescent components confined within the cavity lead to significantly different internal field and reflection characteristics. (Detailed order‐resolved analysis is provided in Section S.5 and Figure S30.)

**FIGURE 6:**
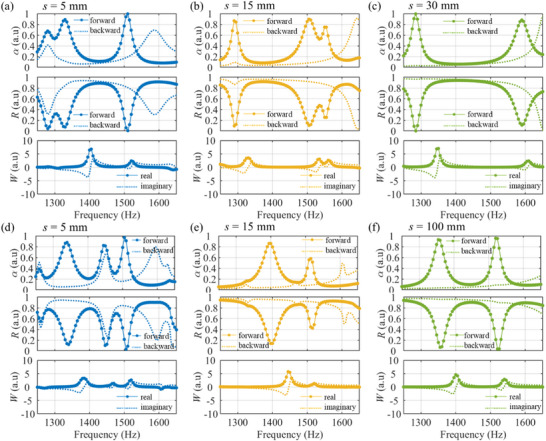
Absorption (top), reflectance (middle) for forward and backward incidence, and the corresponding Willis coupling coefficient *W* (bottom) for different cavity length *s*, shown for bilayer configurations of (a–c) (*m*_1_, *m*_2_) = (4, 2) and (d–f) (3, 2).

Previous studies have described such asymmetric responses in terms of bianisotropic acoustic characteristics, arising from structural asymmetry or nonlocal interactions [40, 41, 42, 43]. In our bilayer system, the evanescent‐wave coupling describes the microscopic interaction where higher‐order diffraction components facilitate the near‐field interaction through the complex scattering events across the bilayer system. To explicitly connect our analysis to the macroscopic bianisotropic perspective, we quantify the asymmetry using the Willis coupling coefficient *W*, evaluated from the scattering parameters including the forward and backward reflection coefficients and the (reciprocal) transmission coefficient. Figure 6a–c presents the absorption, reflectance spectra, and *W* for the (4, 2) and (2, 4) configurations at three cavity lengths (*s* = 5 mm, 15 mm, and 30 mm). Particularly, our analysis reveals that the Willis coupling coefficient *W* is highly sensitive to the cavity length *s*, exhibiting peaks that correspond to the high absorption conditions. Note that the considered bilayer configuration does not strictly satisfy the subwavelength limit (*k*_eff_ *d* ≪ 1) required for standard effective parameter retrieval. Therefore, the retrieved parameters are interpreted as indicators of the asymmetric response rather than intrinsic material properties in this study.

Figure 6d–f presents the absorption, reflectance, and corresponding Willis coupling coefficient for the (3, 2) and (2, 3) configurations, demonstrating that direction‐dependent absorption also arises in odd–even configurations. For the (2, 3) bilayer, a notable difference is that the first metasurface does not generate a fundamental (*n* = 0) component in the cavity, which leads to a distinct absorption profile compared to the reversed configuration. Within the cavity, the acoustic field is dominated by higher diffraction orders (e.g., *n* = ±1 and ±3), and the high absorption is limited to relatively localized peaks associated with evanescent‐wave coupling capable of regenerating a fundamental component (see Section S.5 for broadband absorption performance of the (2, 4) and (2, 3) configurations). Unlike the highly tunable broadband behavior highlighted in Figure 5, these reversed cases exhibit high absorption only over limited frequency ranges.

### 3.3 Realization of Metasurface Units and Experimental Model Validation

To validate the underlying mechanism proposed in this study, we realized the bilayer metasurfaces and conducted full‐wave numerical simulations to evaluate its absorption performance. Each metasurface was designed using a space‐coiling structure, in which the acoustic path within each unit cell is extended via a coiled channel. This design enables the desired phase delay *ϕ*_ij_ $\left(=n_{\mathrm{ij}}^{\mathrm{eff}}k_{0}w\right)$ at the target operating frequency of 1500 Hz, while maintaining a compact overall width *w*. The effective refractive index $n_{\mathrm{ij}}^{\mathrm{eff}}$ of the *j*th unit cell of metasurface *i* can be estimated as 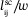, where 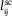 denotes effective acoustic path length inside the coiled channel (see Section S.4 for detailed unit structure). In this study, we designed space‐coiling structures with a fixed channel width of 8 mm and varied channel height 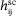 to control the effective path length. Due to the relatively narrow height compared to the unit cell height *a*, end corrections were introduced to account for additional phase delays at the channel openings. To do this, we conducted preliminary simulations on multiple unit cells with different channel lengths and fine‐tuned the design until each unit cell satisfied the target phase delay. Based on this procedure, we obtained sets of unit cells corresponding to (*m*_1_, *m*_2_) = (4, 2), (4, 4), and (3, 2) as summarized in Table S1 of Section S.4. The present prototype is designed at a target operating frequency of 1500 Hz using space‐coiling unit cells. The single‐unit validation measurements were conducted in the 1000–1600 Hz range by the square impedance tube described in Section 5 (first cutoff frequency ∼1700 Hz). The proposed tunable absorption, enabled by evanescent higher‐order diffraction components, is not intrinsically restricted to this frequency range. Since the phase delay depends on the effective acoustic path length, the operating frequency can be readily shifted by scaling the unit‐cell geometry. Furthermore, for practical applications requiring structural compactness, the space‐coiling elements can be replaced by subwavelength resonators (e.g., Helmholtz resonators) [3, 17, 44] to reduce the overall device thickness while maintaining the required phase profiles.

**FIGURE 7:**
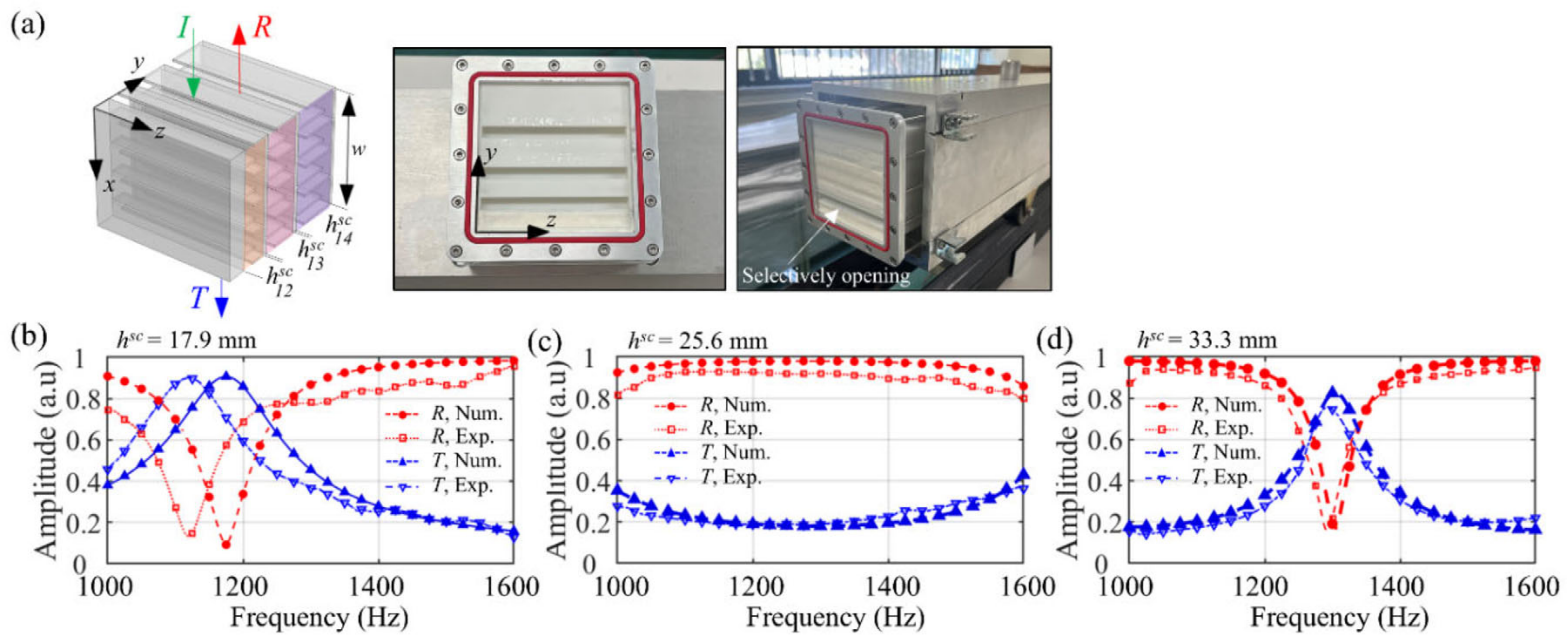
Experimental validation of the numerical model for space‐coiling units. (a) Photograph of the impedance tube setup with a fabricated sample, which contains three distinct unit structures. (b–d) Comparison of simulated and measured reflection and transmission coefficients for each of the three units, which were tested individually by selectively opening their inlets. The units correspond to channel heights of (b) 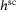
= 17.9 mm, (c) 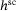
= 25.6 mm, and (d) 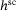
= 33.3 mm.

To validate the numerical model used for the full‐wave simulations, the scattering coefficients of individual space‐coiling units were measured in a square impedance tube and compared with simulation results. Figure 7a shows a fabricated sample and experimental setup. Figure 7b–d presents the simulated and measured scattering parameters for three units with different channel heights 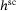 (or different effective refractive index). These three units correspond to the subunits of the *m* = 4 metasurface. The numerical model accurately predicts the resonance frequencies and the magnitude of the scattering coefficients in all cases. For instance, for the single unit with 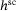
= 17.9 mm (Figure 7b), a resonance is predicted at 1175 Hz and observed experimentally at 1120 Hz (4.7% difference). At this frequency, the numerical model gives *R* = 0.09 and *T* = 0.91, in good agreement with the measurements of 0.13 and 0.90. For the unit of 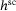
= 25.6 mm, no distinct resonance is observed within the measured frequency range, showing a reflection having a maximum value near 1290 Hz with *R* = 0.98 numerically and 0.92 experimentally as shown in Figure 7c. For the unit of 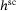
= 33.3 mm, the resonance occurs at 1305 Hz, compared with 1292 Hz in measurement (1.0% difference), with *R* = 0.17 and *T* = 0.83 from the numerical model and 0.15 and 0.76 from the measurements. The discrepancies can be attributed to fabrication tolerances, residual resin inherent to the printing process, and possible interactions between unit structures or fluid–structure coupling that were not fully captured in the numerical simulations. Despite these factors, the numerical model accurately predicts the resonance frequencies and the order of magnitude of the scattering coefficients across cases, indicating that it successfully captures the effective refractive‐index change induced by space‐coiling and dissipative effects in the narrow channels.

### 3.4 Numerical Validation

**FIGURE 8:**
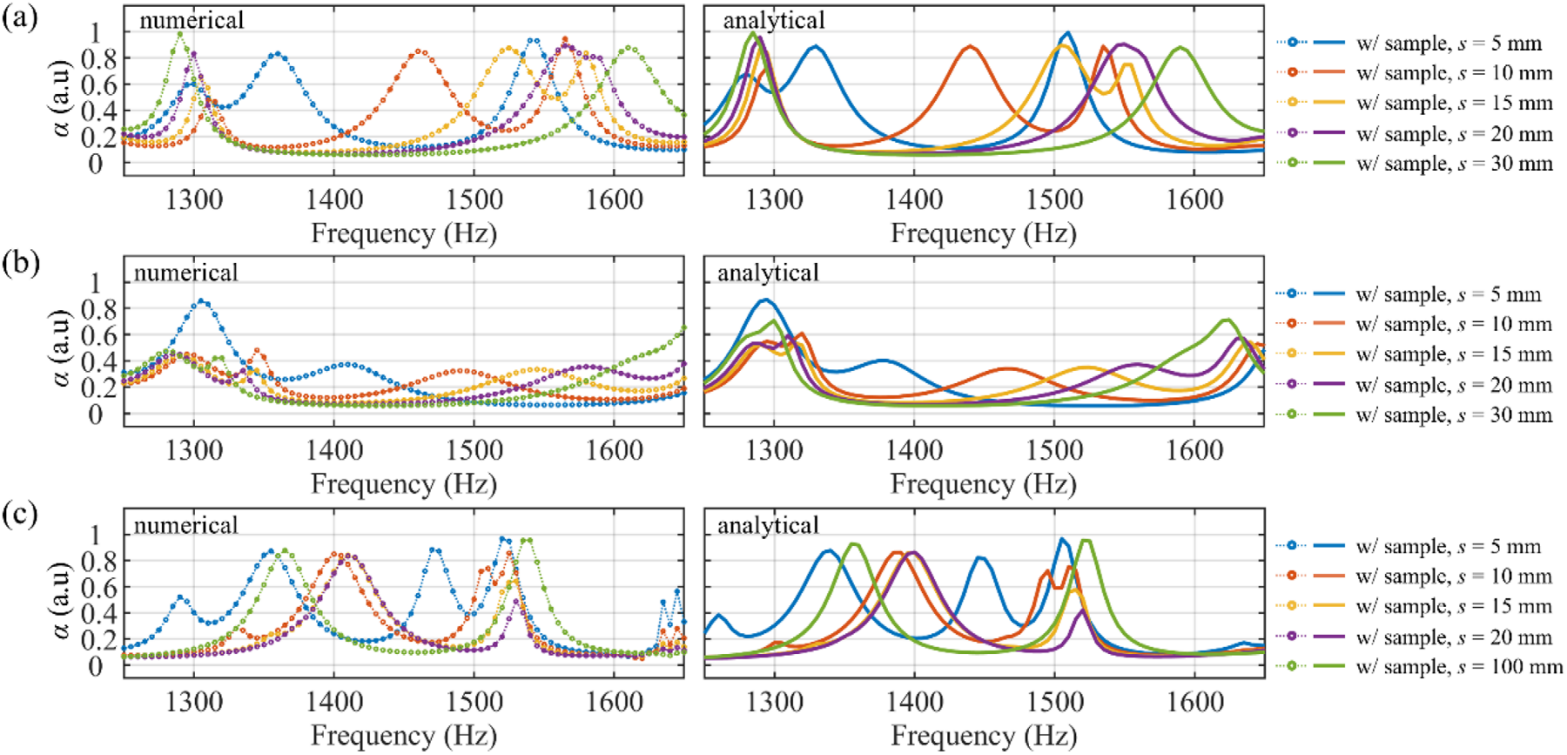
Absorption coefficient of the bilayer metasurface evaluated at various cavity lengths s, obtained from full‐wave numerical simulation (left) and analytical CMT model (right). Configurations shown are (a) (*m*_1_, *m*_2_) = (4, 2), (b) (4, 4), and (c) (3, 2).

We then performed full‐wave simulations using the realized (*m*_1_, *m*_2_) = (4, 2) bilayer metasurfaces and compared their performance with analytical predictions. Figure 8a shows the absorption coefficient obtained from full‐wave simulations (left), compared with the CMT model results (right). The simulations demonstrate that the (4, 2) configuration achieves strong absorption exceeding 85% for each cavity length. At *s* = 5, 10, and 15 mm, two distinct absorption peaks are observed —for example, at 1360 and 1540 Hz for *s* = 5 mm. At *s* = 20 mm, two peaks are observed close to 1565 Hz, whereas at *s* = 30 mm, only a single absorption peak appears at 1610 Hz. The high absorption across these cases confirms the effectiveness of evanescent coupling in trapping acoustic energy within the bilayer system and suppressing far‐field transmission. A slight frequency shift is observed between the numerical and analytical peaks (e.g., 1540 and 1510 Hz at *s* = 5 mm), which can be attributed to differences in acoustic impedance of the realized structure and the effect of thermo‐viscous losses. Note that each single metasurface absorbs a small part of the incident energy, primarily due to attenuation from internal reflections in the coiled channels. However, when the two metasurfaces are combined with a small cavity, the overall absorption can be tuned from under 20% up to 95% by adjusting cavity length *s*. This significant enhancement confirms the effectiveness of evanescent coupling.

**FIGURE 9:**
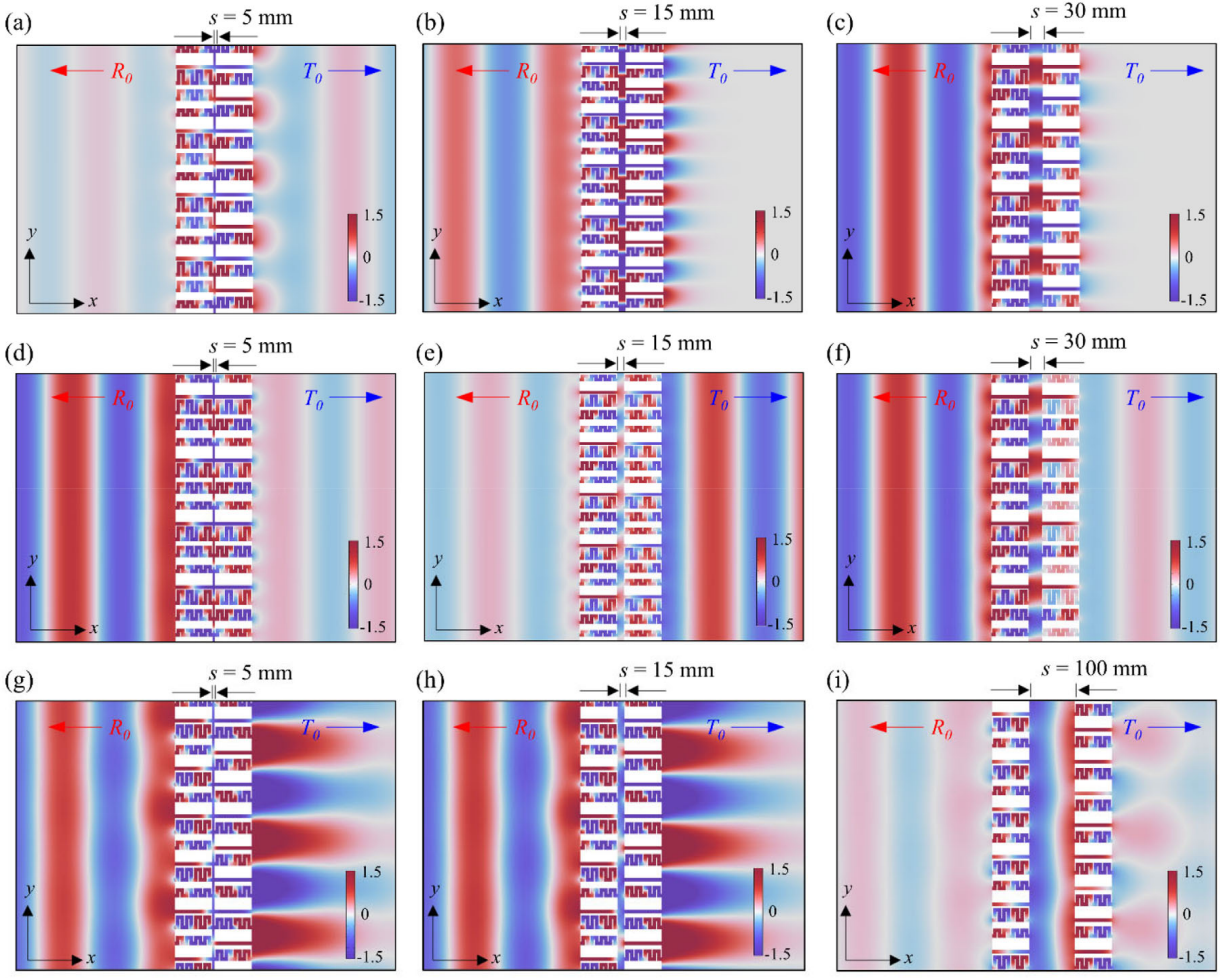
Pressure field distributions of scattered waves at 1540 Hz for the bilayer metasurface. (a–c) Scattering behavior of the (4, 2) configuration with cavity lengths *s* = 5, 15, and 30 mm, respectively. High sound absorption is observed at *s* = 5 mm, where both reflected and transmitted waves are effectively suppressed due to strong evanescent coupling. (d–f) Corresponding field distributions for the (4, 4) configuration at the sample cavity lengths, exhibiting relatively stronger scattered waves and limited suppression of the fundamental mode. (g–i) Scattered field for the (3, 2) configuration with cavity lengths *s* = 5, 15, and 100 mm, respectively. Maximum absorption is observed at *s* = 100 mm, which approximately corresponds to half the wavelength, where the 0th‐order components are confined within the cavity.

Figure 9a–c illustrates the pressure field distributions of the scattered waves at 1540 Hz for three different cavity lengths. At the optimal cavity length (*s* = 5 mm, Figure 9a), weak scattered waves are observed in either the upstream or downstream region, indicating maximal energy absorption in the system. In contrast, at off‐optimal separations (*s* = 15 mm and 30 mm, shown in Figure 9b,c), strong reflected waves appear upstream, resulting in lower absorption performance. Nevertheless, even in these conditions, no transmitted wave is observed in the downstream region. The second metasurface still effectively suppresses the 0th‐order transmission. These results are consistent with the theoretical mechanism discussed earlier.

In contrast, Figure 8b,c presents the numerical results for the (4, 4) and (3, 2) configuration with varying cavity lengths. As predicted analytically, the absorption coefficient of the (4, 4) configuration remains below 50%, except at specific local resonances, and its dependence on the cavity length *s* is relatively weak. To further illustrate the wave behavior, Figure 9d–f shows the scattered pressure fields of the (4, 4) configuration for three different values of *s*. Compared to the (4, 2) configuration, the (4, 4) case exhibits stronger scattered fields. Notably, near the optimal value of *s* = 15 mm, the transmitted wave becomes more dominant, consistent with the CMT prediction shown in Figure 4d. In this symmetric configuration, evanescent‐wave coupling allows the 0th‐order mode to propagate downstream, thereby limiting the absorption performance. This behavior contrasts with the (4, 2) case, where the asymmetric configuration more effectively suppresses the far‐field transmission and enables enhanced absorption through the strong evanescent coupling mechanism. For the (3, 2) configuration, the high absorption coefficient exceeding 85% is observed at 1520, 1400, and 1540 Hz for cavity length *s* = 5, 10, and 100 mm, respectively. As predicted by the CMT model (Figure 4f), significant fundamental (0th‐order) components appear within the cavity. Specifically, at *s* = 100 mm (close to half the wavelength at the operating frequency), both the reflected and transmitted waves are effectively suppressed at the absorption peak. This is presented in Figure 9i, where the 0th‐order components are effectively diffracted within the cavity. Although the (3, 2) configuration achieves high absorption peaks at small cavity lengths *s*, it exhibits only localized peaks confined within a relatively narrow frequency range, in contrast to the more robust performance observed in the (4, 2) configuration.

## 4 Conclusion

This study demonstrated a tunable mechanism for sound absorption enabled by evanescent‐wave coupling in an asymmetric bilayer metasurface system. By simply adjusting the sub‐wavelength air cavity between the layers, the system transitions from a reflective to a highly absorptive state, achieving near‐perfect absorption. To investigate the tunability of absorption, we systematically explored various bilayer configurations (*m*_1_, *m*_2_) and analytically examined the role of evanescent waves generated by the phase‐gradient metasurfaces. This analysis revealed that strong evanescent‐wave coupling and high‐contrast absorption are achieved in specific asymmetric configurations, a mechanism critically determined by the cavity length and the integer parity of each metasurface layer.

Theoretical and numerical analyses show that a bilayer composed of two distinct metasurface designs achieves high sound absorption at a sub‐wavelength cavity length. In these optimal cases, both the fundamental order of reflection and transmission are strongly suppressed, leading to efficient confinement of acoustic energy. In contrast, symmetric configurations exhibit limited performance due to partial transmission of the fundamental mode, which results from reciprocal scattering between identical layers. The proposed mechanism was validated through full‐wave simulations using a space‐coiling design, with the numerical model being verified by experimental measurements of single unit cells. The bilayer system exhibited robust broadband performance, maintaining high absorption tunability across a wide frequency range.

A key advantage of this configuration is its ability to control acoustic performance simply by tuning the cavity length, without modifying the unit‐cell geometry. Unlike many existing tunable acoustic metasurfaces that require internal reconfiguration or active elements, the proposed approach offers high tunability while maintaining a simple structural design. These findings pave the way for next‐generation acoustic systems, including tunable noise barriers, broadband absorbers, and reconfigurable acoustic devices, that can dynamically adapt to changing environments. Future work should involve the experimental validation of the bilayer metasurface system and the development of practical mechanisms for integrating this tunability into functional devices.

## 5 Experimental Method

To validate the numerical model for the space‐coiling structures, the scattering coefficients of single unit cells were measured using a square impedance tube with a 100  × 100 mm^2^ cross‐section (first cutoff frequency is approximately 1700 Hz). As shown in Figure 7a, the space‐coiling units corresponding to the 2nd–4th units of the *m* = 4 metasurface were fabricated by stereolithography (SLA). Note that the geometry of each unit follows Table S1 in the Section S.4. To separate incident, reflected and transmitted waves, two microphones, GRAS type 46AD, were placed 50 mm apart upstream and downstream of the specimen, respectively. A random excitation in 1–2 kHz was generated by a function generator (EDU33212A, Keysight Corp.), and sound measurements were performed at a sampling rate of 50 kHz using a data acquisition module (NI‐9234, National Instruments). Auto‐ and cross‐spectra of the measured signals were averaged over 30 s, and reflection and transmission coefficients were calculated by decomposing the forward and backward propagating wave components following the ASTM procedure [45]. To isolate the response of a single space‐coiling unit, its inlet was selectively opened while the other two units were sealed with tape. This ensured that the upstream and downstream ducts were connected only through the target unit, preventing sound transmission through the other channels.

## Conflicts of Interest

The authors declare no conflicts of interest.

## Supporting information




**Supporting File**: advs74181‐sup‐0001‐SuppMat.pdf.

## Data Availability

The data that support the findings of this study are available from the corresponding author upon reasonable request.
