# Supplementary material for: Tunable Sound Absorption via Evanescent‐Wave Coupling in Asymmetric Bilayer Metasurfaces
Source: Adv Sci (Weinh). 2026 Feb 6;13(20):e22261. doi: 10.1002/advs.202522261 (PMC13067807; doi:10.1002/advs.202522261)
Supplement: Supplementary file 1 — Supporting File: advs74181‐sup‐0001‐SuppMat.pdf. [file ADVS-13-e22261-s001.pdf]

Supporting Information for the article:
Tunable sound absorption via evanescent-wave coupling in asymmetric bilayer metasurfaces

Pyung Sik Ma[1] and Hyung Jin Lee,[2]

[1] *Department of Mechanical Engineering, Gachon University, Seongnam 13120, Republic of Korea*

[2] *Acoustics, Ultrasound, and Vibration Metrology Group, Korea Research Institute of Standards and Science, Daejeon 34113, Republic of Korea*

S.1 Amplitudes of scattering parameters for bilayer metasurfaces

This section provides detailed amplitudes of the diffraction orders for all bilayer metasurface combinations with m_1 and m_2 ranging from 2 to 6, as calculated by the coupled-mode theory (CMT) at 1500 Hz. The results are organized into 5 pair groups based on the integer parity of m_1 and m_2. Here, R_n, T_n, F_n, and B_n denote the amplitudes of the nth reflected, transmitted, forward, and backward components (inside the cavity), respectively, computed using coupled-mode theory (CMT) at 1500 Hz.

S.1.1 Pair group 1 ($m_1 = m_2$)

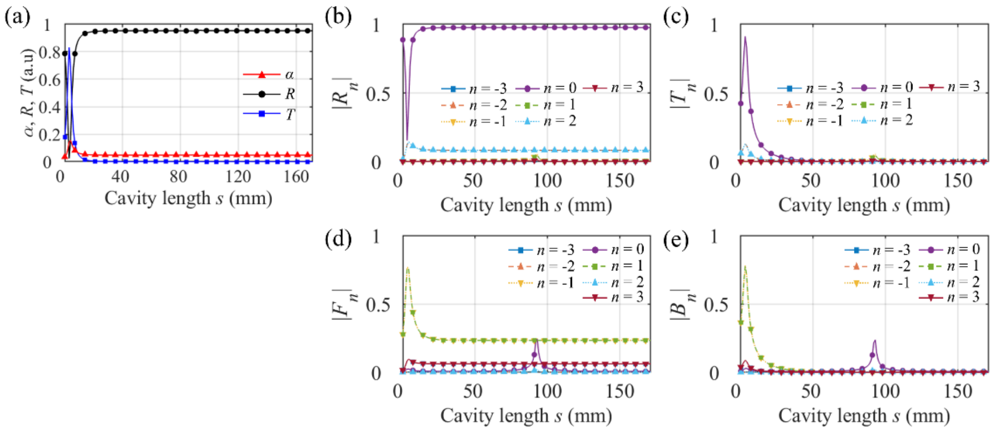

Fig. S1. (a) Absorption (triangles), reflectance (circles), and transmittance (squares) as a function of cavity length s at 1500 Hz for the $(m_1, m_2) = (2, 2)$ bilayer configuration. (b-e) Amplitudes of R_n, T_n, F_n, and B_n.

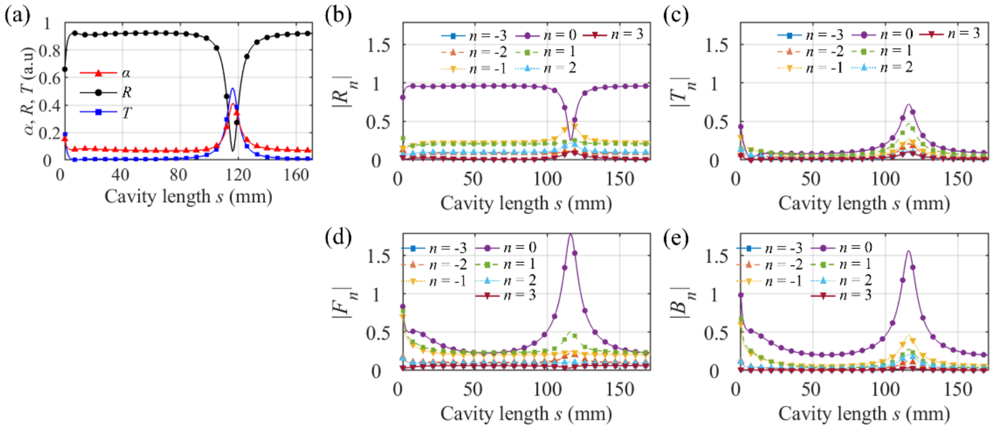

Fig. S2. (a) Absorption (triangles), reflectance (circles), and transmittance (squares) as a function of cavity length s at 1500 Hz for the (m_1, m_2) = (3, 3) bilayer configuration. (b-e) Amplitudes of R_n, T_n, F_n, and B_n.

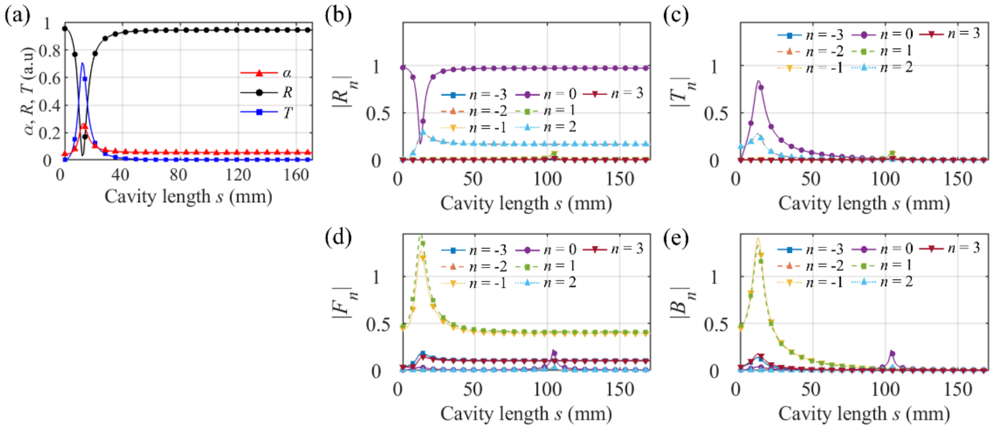

Fig. S3. (a) Absorption (triangles), reflectance (circles), and transmittance (squares) as a function of cavity length s at 1500 Hz for the (m_1, m_2) = (4, 4) bilayer configuration. (b-e) Amplitudes of R_n, T_n, F_n, and B_n.

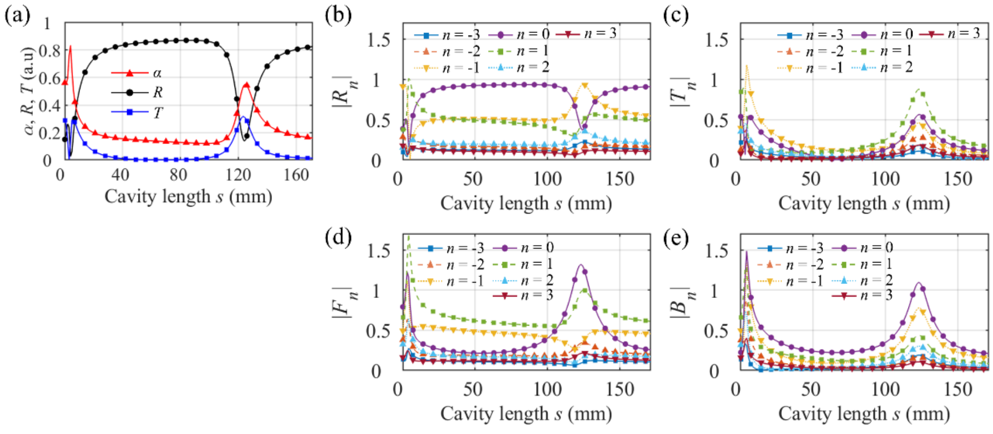

Fig. S4. (a) Absorption (triangles), reflectance (circles), and transmittance (squares) as a function of cavity length s at 1500 Hz for the (m_1, m_2) = (5, 5) bilayer configuration. (b-e) Amplitudes of R_n, T_n, F_n, and B_n.

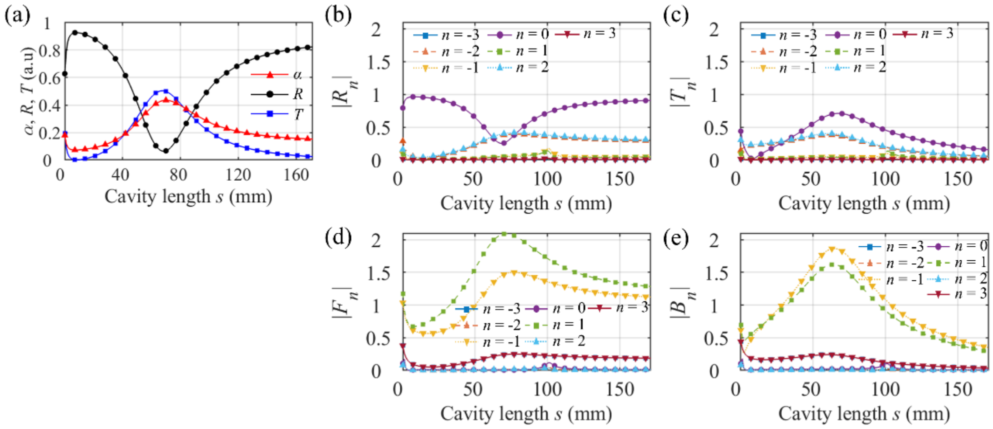

Fig. S5. (a) Absorption (triangles), reflectance (circles), and transmittance (squares) as a function of cavity length s at 1500 Hz for the (m_1, m_2) = (6, 6) bilayer configuration. (b-e) Amplitudes of R_n, T_n, F_n, and B_n.

S.1.2 Pair group 2 (both m_1, m_2 odd but $m_1 \neq m_2$)

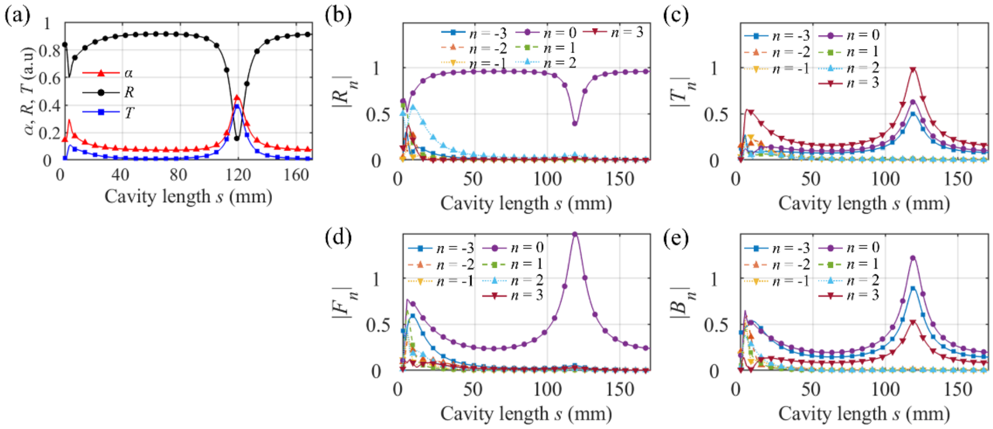

Fig. S6. (a) Absorption (triangles), reflectance (circles), and transmittance (squares) as a function of cavity length s at 1500 Hz for the $(m_1, m_2) = (3, 5)$ bilayer configuration. (b-e) Amplitudes of R_n, T_n, F_n, and B_n.

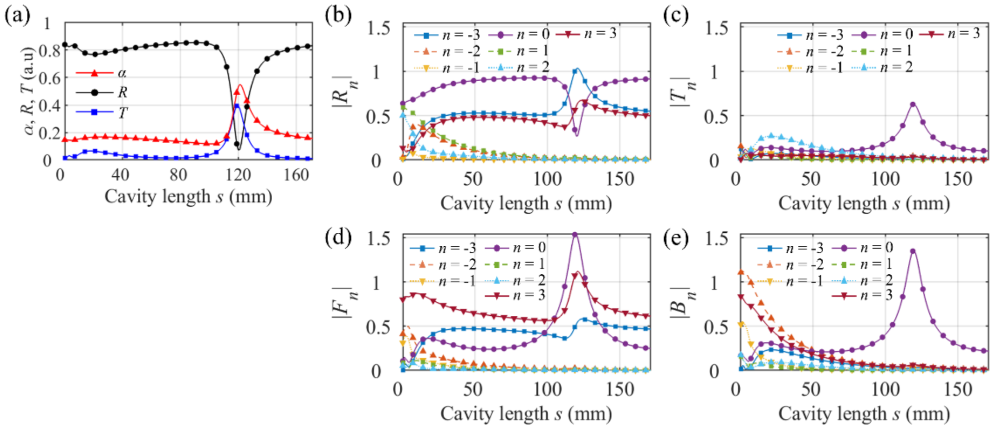

Fig. S7. (a) Absorption (triangles), reflectance (circles), and transmittance (squares) as a function of cavity length s at 1500 Hz for the $(m_1, m_2) = (5, 3)$ bilayer configuration. (b-e) Amplitudes of R_n, T_n, F_n, and B_n.

S.1.3 Pair group 3 (m_1 odd, m_2 even)

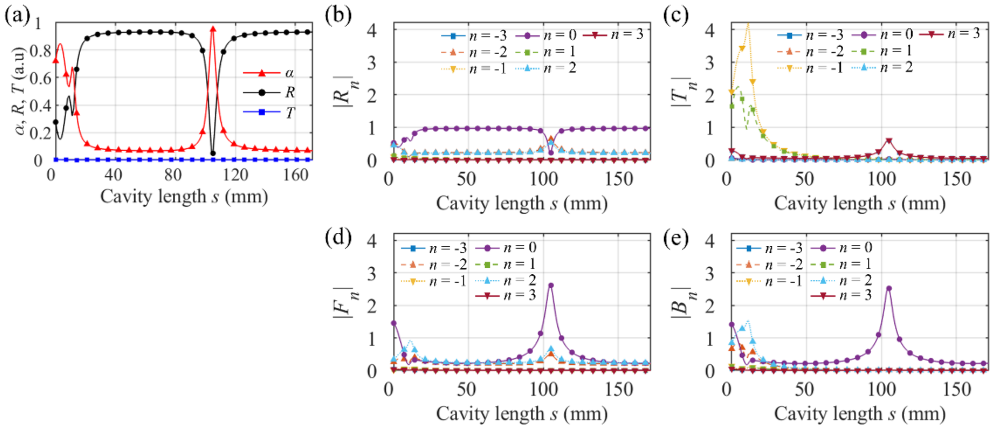

Fig. S8. (a) Absorption (triangles), reflectance (circles), and transmittance (squares) as a function of cavity length s at 1500 Hz for the (m_1, m_2) = (3, 2) bilayer configuration. (b-e) Amplitudes of R_n, T_n, F_n, and B_n.

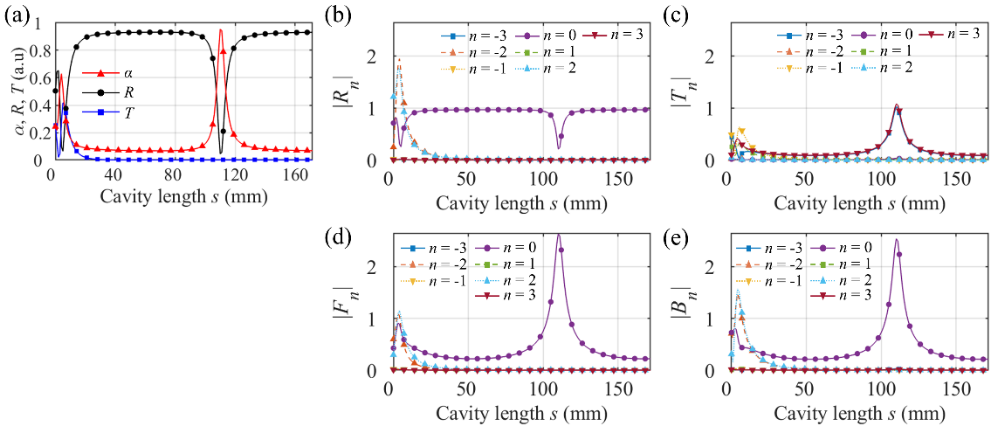

Fig. S9. (a) Absorption (triangles), reflectance (circles), and transmittance (squares) as a function of cavity length s at 1500 Hz for the (m_1, m_2) = (3, 4) bilayer configuration. (b-e) Amplitudes of R_n, T_n, F_n, and B_n.

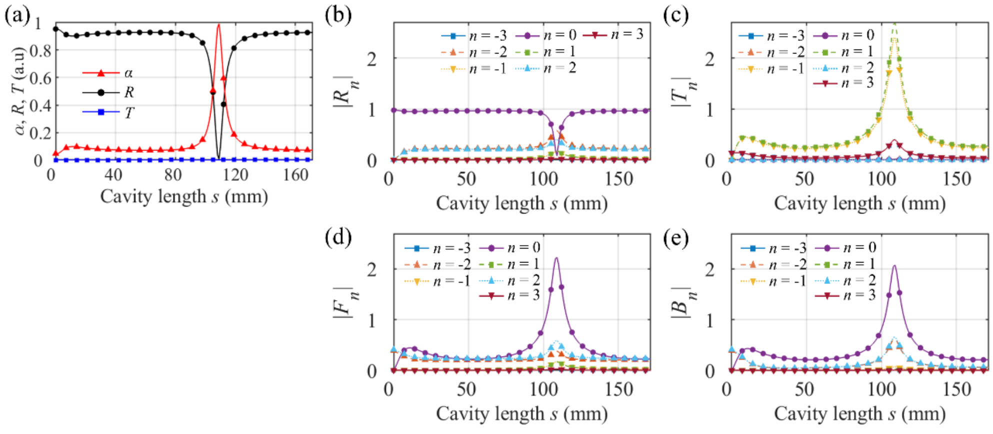

Fig. S10. (a) Absorption (triangles), reflectance (circles), and transmittance (squares) as a function of cavity length s at 1500 Hz for the $(m_1, m_2) = (3, 6)$ bilayer configuration. (b-e) Amplitudes of R_n, T_n, F_n, and B_n.

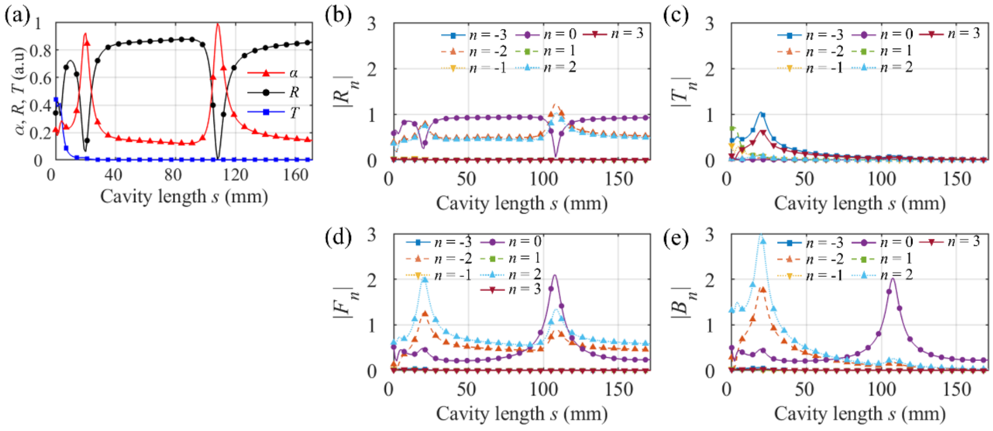

Fig. S11. (a) Absorption (triangles), reflectance (circles), and transmittance (squares) as a function of cavity length s at 1500 Hz for the $(m_1, m_2) = (5, 2)$ bilayer configuration. (b-e) Amplitudes of R_n, T_n, F_n, and B_n.

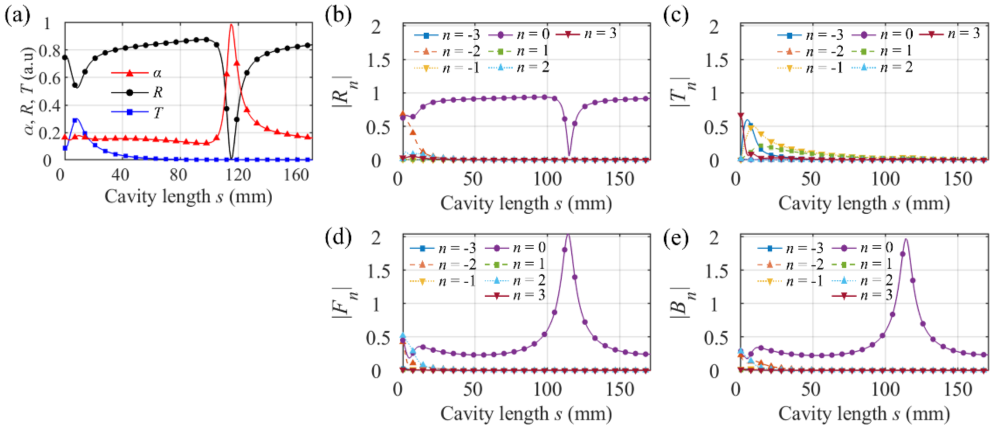

Fig. S12. (a) Absorption (triangles), reflectance (circles), and transmittance (squares) as a function of cavity length s at 1500 Hz for the (m_1, m_2) = (5, 4) bilayer configuration. (b-e) Amplitudes of R_n, T_n, F_n, and B_n.

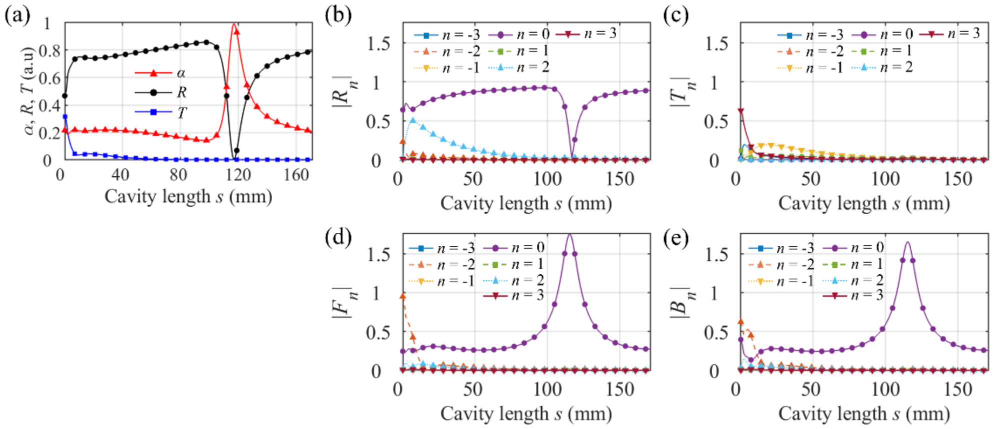

Fig. S13. (a) Absorption (triangles), reflectance (circles), and transmittance (squares) as a function of cavity length s at 1500 Hz for the (m_1, m_2) = (5, 6) bilayer configuration. (b-e) Amplitudes of R_n, T_n, F_n, and B_n.

S.1.4 Pair group 4 (both m_1, m_2 even but $m_1 \neq m_2$)

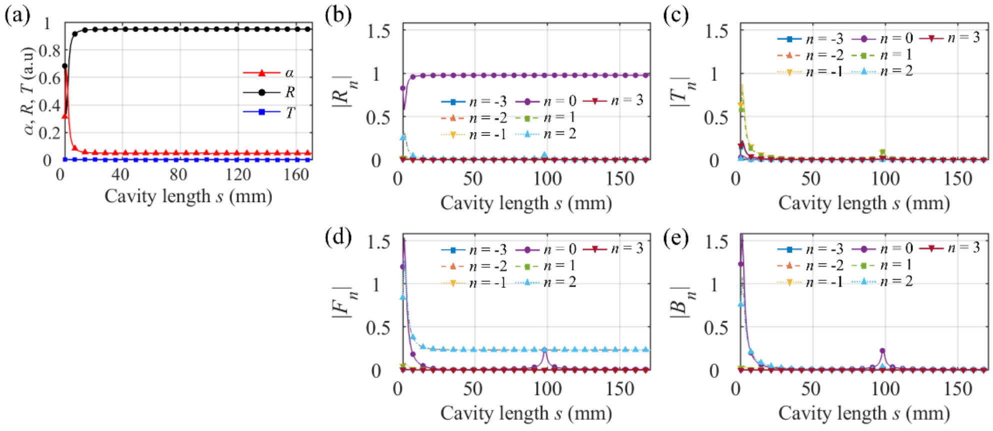

Fig. S14. (a) Absorption (triangles), reflectance (circles), and transmittance (squares) as a function of cavity length s at 1500 Hz for the (m_1, m_2) = (2, 4) bilayer configuration. (b-e) Amplitudes of R_n, T_n, F_n, and B_n.

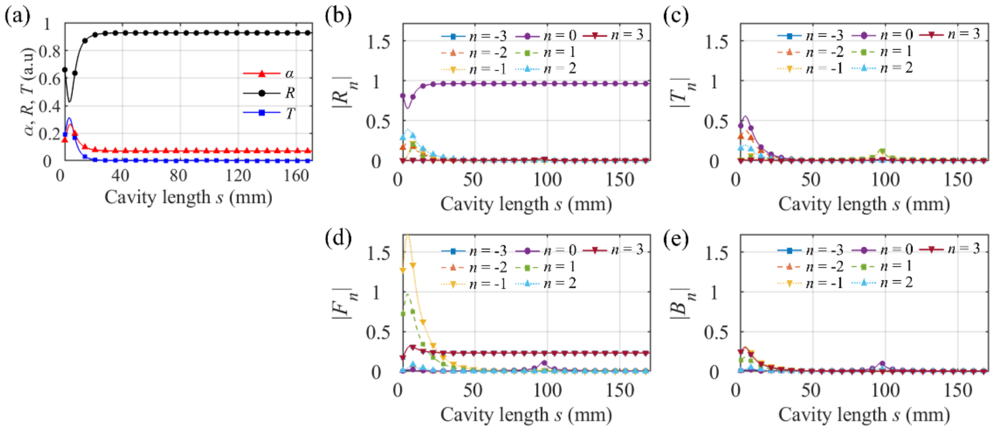

Fig. S15. (a) Absorption (triangles), reflectance (circles), and transmittance (squares) as a function of cavity length s at 1500 Hz for the (m_1, m_2) = (2, 6) bilayer configuration. (b-e) Amplitudes of R_n, T_n, F_n, and B_n.

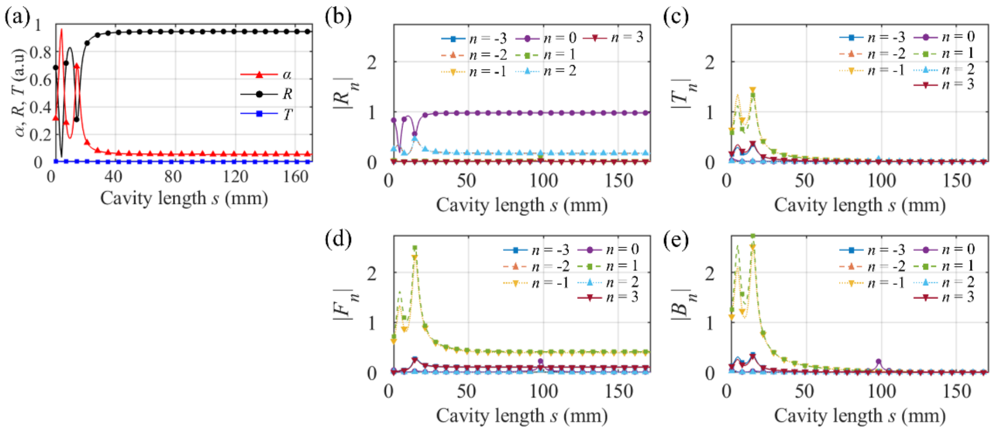

Fig. S16. (a) Absorption (triangles), reflectance (circles), and transmittance (squares) as a function of cavity length s at 1500 Hz for the $(m_1, m_2) = (4, 2)$ bilayer configuration. (b-e) Amplitudes of R_n, T_n, F_n, and B_n.

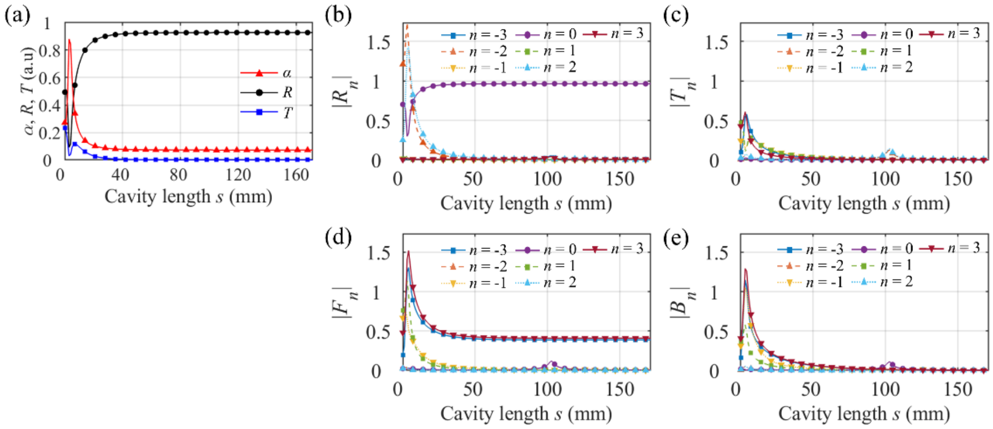

Fig. S17. (a) Absorption (triangles), reflectance (circles), and transmittance (squares) as a function of cavity length s at 1500 Hz for the $(m_1, m_2) = (4, 6)$ bilayer configuration. (b-e) Amplitudes of R_n, T_n, F_n, and B_n.

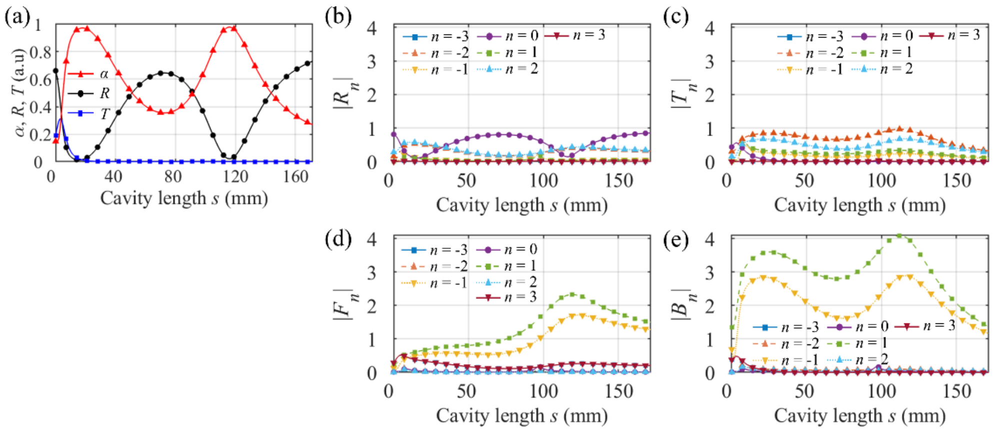

Fig. S18. (a) Absorption (triangles), reflectance (circles), and transmittance (squares) as a function of cavity length *s* at 1500 Hz for the (m_1, m_2) = (6, 2) bilayer configuration. (b-e) Amplitudes of R_n, T_n, F_n, and B_n.

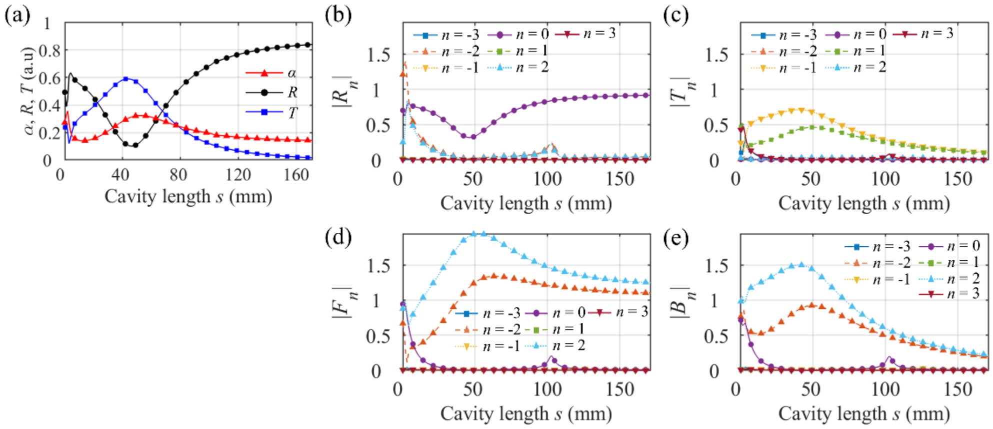

Fig. S19. (a) Absorption (triangles), reflectance (circles), and transmittance (squares) as a function of cavity length *s* at 1500 Hz for the (m_1, m_2) = (6, 4) bilayer configuration. (b-e) Amplitudes of R_n, T_n, F_n, and B_n.

S.1.5 Pair group 5 (m_1 even, m_2 odd)

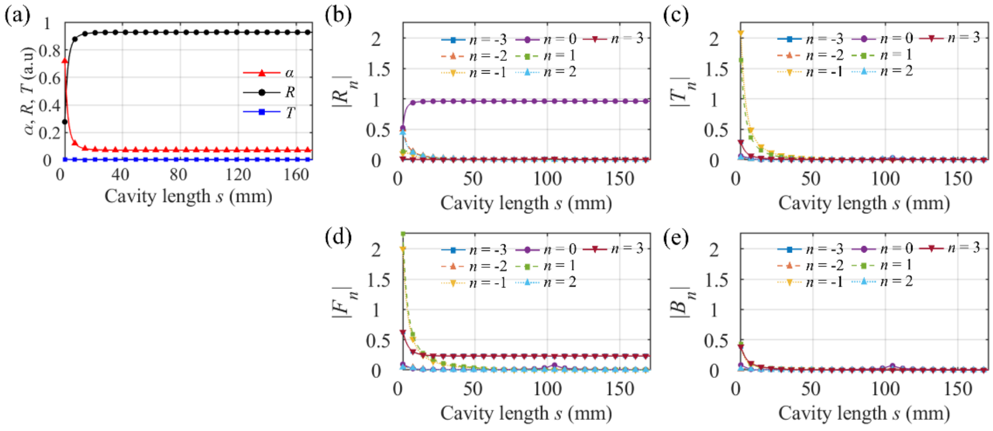

Fig. S20. (a) Absorption (triangles), reflectance (circles), and transmittance (squares) as a function of cavity length s at 1500 Hz for the (m_1, m_2) = (2, 3) bilayer configuration. (b-e) Amplitudes of R_n, T_n, F_n, and B_n.

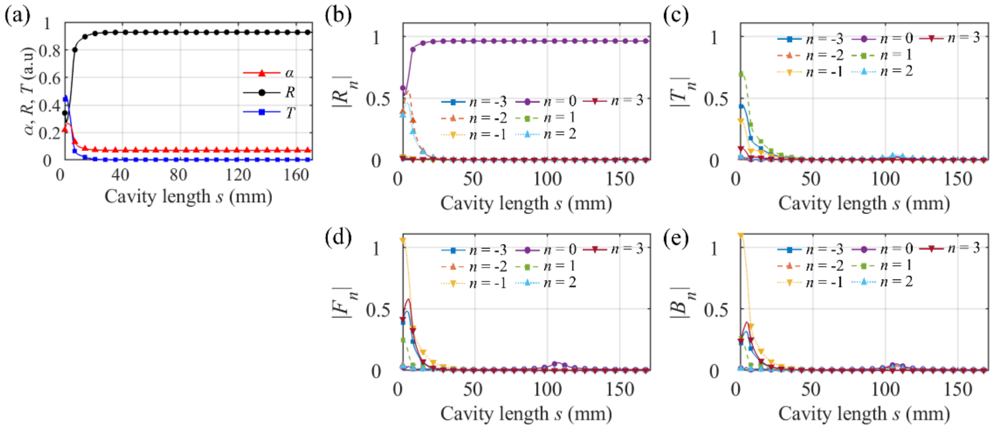

Fig. S21. (a) Absorption (triangles), reflectance (circles), and transmittance (squares) as a function of cavity length s at 1500 Hz for the (m_1, m_2) = (2, 5) bilayer configuration. (b-e) Amplitudes of R_n, T_n, F_n, and B_n.

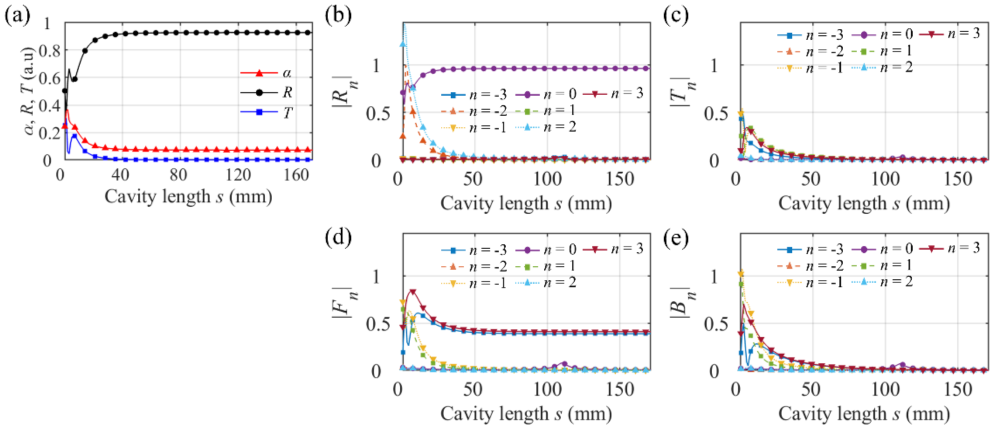

Fig. S22. (a) Absorption (triangles), reflectance (circles), and transmittance (squares) as a function of cavity length s at 1500 Hz for the (m_1, m_2) = (4, 3) bilayer configuration. (b-e) Amplitudes of R_n, T_n, F_n, and B_n.

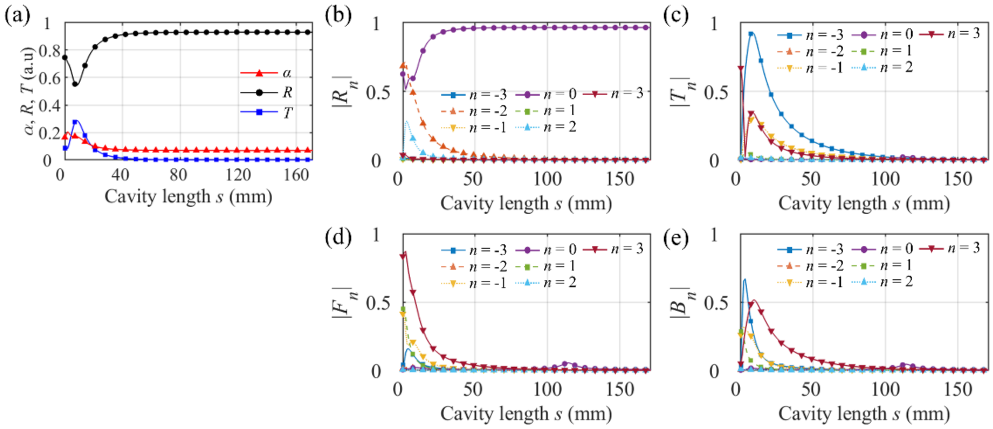

Fig. S23. (a) Absorption (triangles), reflectance (circles), and transmittance (squares) as a function of cavity length s at 1500 Hz for the (m_1, m_2) = (4, 5) bilayer configuration. (b-e) Amplitudes of R_n, T_n, F_n, and B_n.

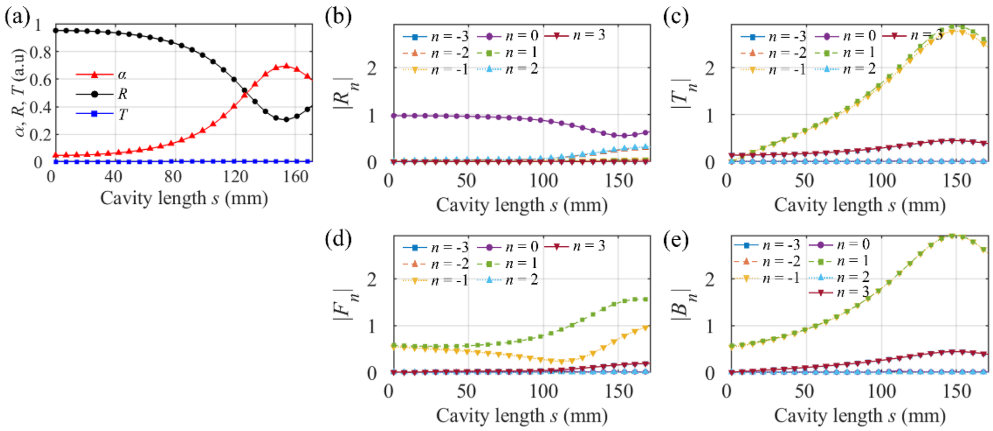

Fig. S24. (a) Absorption (triangles), reflectance (circles), and transmittance (squares) as a function of cavity length s at 1500 Hz for the (m_1, m_2) = (6, 3) bilayer configuration. (b-e) Amplitudes of R_n, T_n, F_n, and B_n.

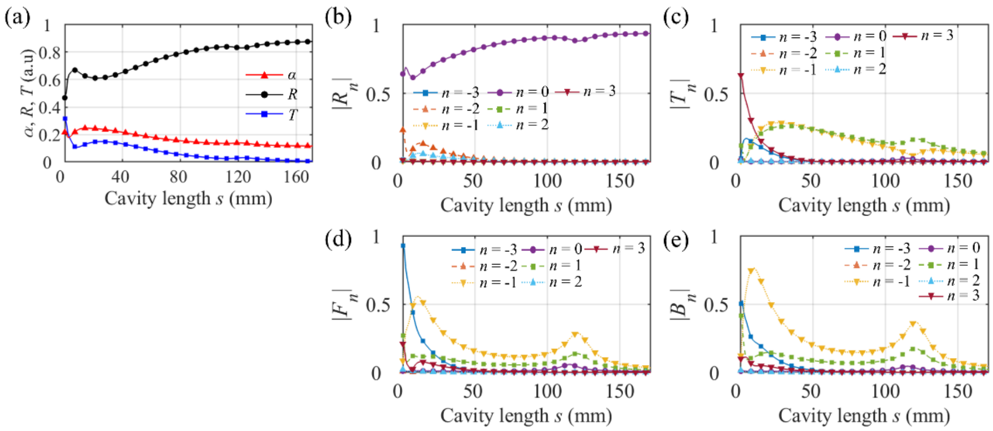

Fig. S25. (a) Absorption (triangles), reflectance (circles), and transmittance (squares) as a function of cavity length s at 1500 Hz for the (m_1, m_2) = (6, 5) bilayer configuration. (b-e) Amplitudes of R_n, T_n, F_n, and B_n.

S.2 Sound scattering by single-layer metasurfaces

To understand the behavior of the bilayer system, it is helpful to examine the scattering characteristics of each individual metasurface. Using the CMT model, we evaluate the scattering parameters for single metasurface with m = 2, 3, and 4 across various diffraction orders when excited by a normally incident plane wave (n = 0) or by individual evanescent modes ($n \neq 0$). Figure S26 shows the order-resolved scattering for single metasurfaces with m = 4 and m = 2. As shown in Fig. S26(a), an m = 4 metasurface reflects nearly all incident energy into the fundamental 0th-order wave (purple bars). The ±1 diffraction orders do transmit through this metasurface shown in the right panel of Fig. S26(a), but transverse wave number exceeds the free-space wave number, so they remain evanescent and cannot propagate into the far field. An m = 2 metasurface (Fig. S26(b)) similarly reflects most energy into the 0th order, while the ±2-order components are transmitted. These ±2 orders are also evanescent, just as the metasurface of m = 4. In summary, each metasurface with an even number of unit cells by itself (whether m = 4 or m = 2) strongly reflects incident wave, and only higher diffraction orders are transmitted through the single metasurface, which is evanescent and confined near the surface of metasurface.

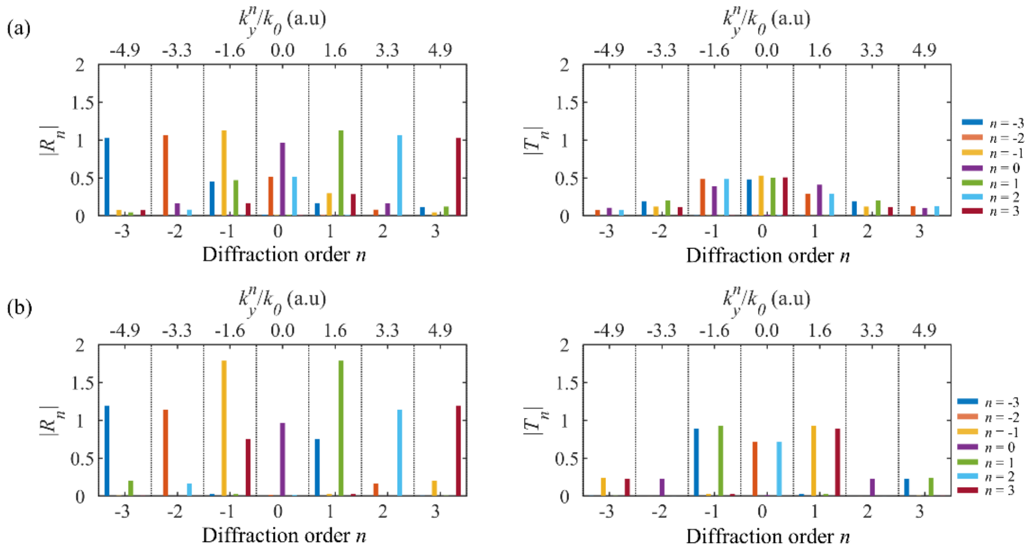

Fig. S26. Diffraction order components for single metasurfaces: reflected waves (left) and transmitted waves (right) when excited by specific diffraction order components. Results are shown for (a) m = 4 and (b) m = 2, evaluated using a supercell of m_s = 4 unit cells. Each bar in the same color represents the reflected and transmitted components when a given diffraction order (n = 0, ±1, ±2, ±3) is incident on the metasurface.

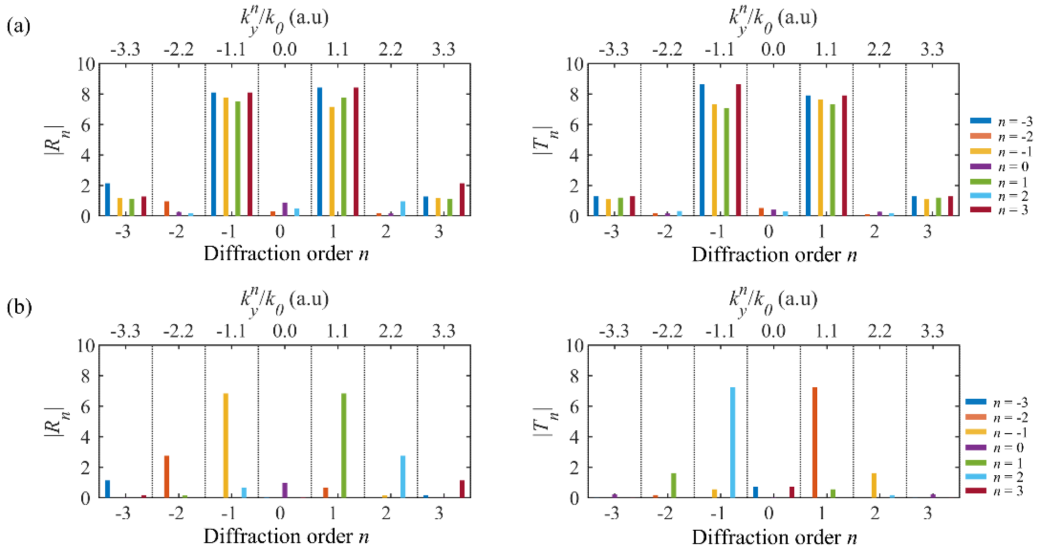

Fig. S27. Diffraction order components for single metasurfaces: Reflected (left) and transmitted (right) wave components under excitation by individual diffraction orders (n = 0, ±1, ±2, ±3). Results are shown for (a) m = 3 and (b) m = 2, evaluated using a supercell of m_s = 6 unit cells. Bars in the same color represent the reflected and transmitted components corresponding to each incident diffraction order.

S.3 Amplitudes of internal waves within unit cells of metasurfaces

This section provides the amplitudes of the forward (a_j, c_j) and backward (b_j, d_j) propagating modes within each unit cell of (m_1, m_2) = (4, 2) bilayer metasurfaces. These plots illustrate which unit cells are most strongly excited at the frequencies corresponding to the high-absorption branches shown in Fig. 5.

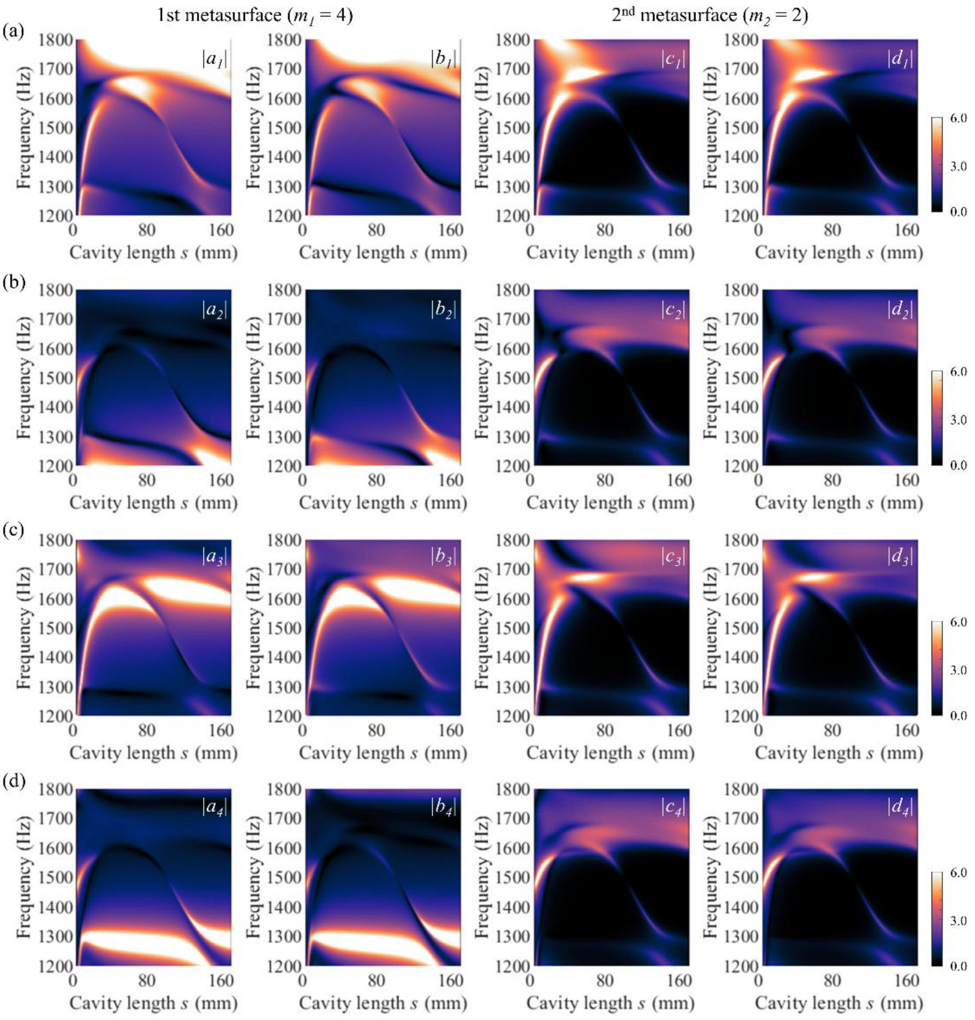

Fig. S28. Amplitudes of the internal wave (|a_j|, |b_j|, |c_j|, |d_j|) within each unit cell of the (m_1, m_2) = (4, 2) bilayer configuration, plotted as a function of frequency and cavity length s. The results show that the two distinct absorption branches observed at sub-wavelength s correspond to the excitation of different sets of unit cell modes.

S.4 Numerical model for full-wave simulation of bilayer metasurfaces

To validate the absorption performance of the bilayer metasurfaces evaluated by the analytical model, we conducted full-wave finite element simulations using COMSOL Multiphysics. As shown in Fig. S29, a numerical model that contains a supercell of the bilayer metasurface was constructed. Periodic boundary condition in the y-direction was applied along the boundaries of the model to reflect coupling between adjacent supercells. The detailed geometry of each unit cell (including the internal space-coiling channel that yields the desired phase delay) was included. Hard-wall boundary conditions were applied to the internal channels of each unit cell, ensuring that coupling between unit cells occurs only through the acoustic fields and not through structural domains. Perfectly matched layers (PMLs) were used to eliminate reflections from the domain boundaries. The mesh size of the finite element model was refined such that the maximum element size was less than $\lambda_0/10$ at the target frequency of 1500 Hz. The scattered field from the bilayer metasurfaces was calculated for a normally incident plane wave ($\theta_i = 0$) by varying the cavity length s between the metasurfaces. The absorption coefficient of the bilayer metasurface was evaluated by subtracting the normalized acoustic energies of the reflected and transmitted waves from that of the incident wave.

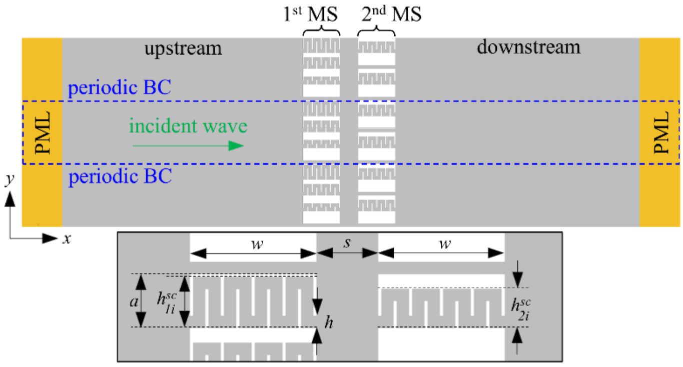

Fig. S29. The analysis domain and boundary conditions used in the full-wave numerical analysis of the bilayer metasurface. The inset shows the detailed geometry of a unit cell designed using a space-coiling structure.

Table S1. Geometric parameters of the bilayer metasurfaces

a (mm)		32.0		
w (mm)		102.0		
h (mm)		8.0		
		$(m_1, m_2) = (4, 2)$	$(m_1, m_2) = (4, 4)$	$(m_1, m_2) = (3, 2)$
1st MS (m_1) (mm)	h^{sc}_{11}	8.0	8.0	8.0
	h^{sc}_{12}	17.9	17.9	20.4
	h^{sc}_{13}	25.6	25.6	30.7
	h^{sc}_{14}	33.3	33.3	8.0
	h^{sc}_{15}	-	-	20.4
	h^{sc}_{16}	-	-	30.7
2nd MS (m_2) (mm)	h^{sc}_{21}	8.0	8.0	8.0
	h^{sc}_{22}	25.6	17.9	25.6
	h^{sc}_{23}	8.0	8.0	8.0
	h^{sc}_{24}	25.6	33.3	25.6
	h^{sc}_{25}	-	-	8.0
	h^{sc}_{26}	-	-	25.6

S.5 Sound scattering under backward incidence

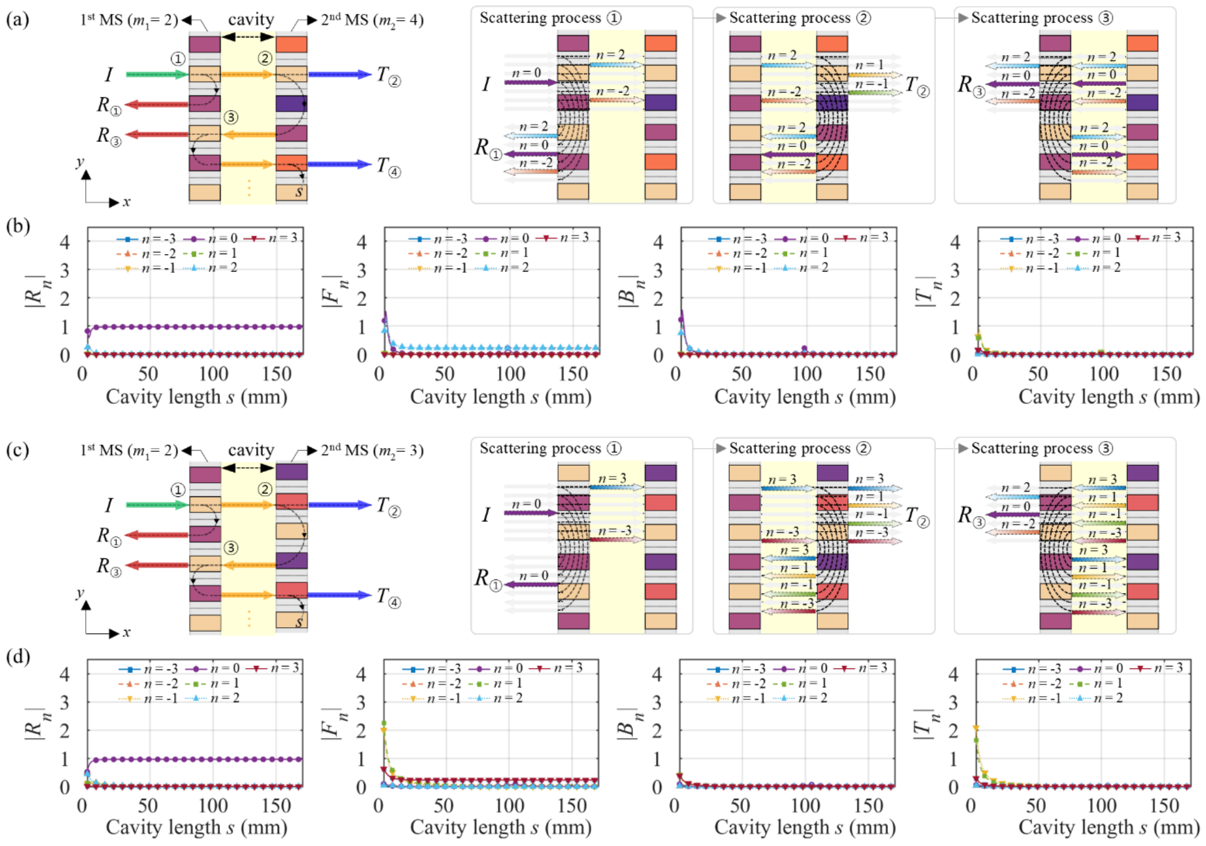

Fig. S30. Order-resolved analysis of the sound absorption mechanism for bilayer metasurfaces. Schematic illustrations of the successive scattering events (processes ①, ②, ③) for (a) (m_1, m_2) = (2, 4) and (c) (2, 3), which correspond to backward-incidence cases for (m_1, m_2) = (4, 2) and (3, 2) respectively. (b,d) Amplitudes of the reflected ($|R_n|$), transmitted ($|T_n|$), forward ($|F_n|$), and backward ($|B_n|$) wave components as a function of the cavity length s at 1500 Hz for the corresponding configurations. Note that although the first metasurface is identical in both cases (m_1 = 2), the diffraction order n correspond to different wave numbers in the two cases due to the different supercell period m_s, determined by the least common multiple with the second metasurface.

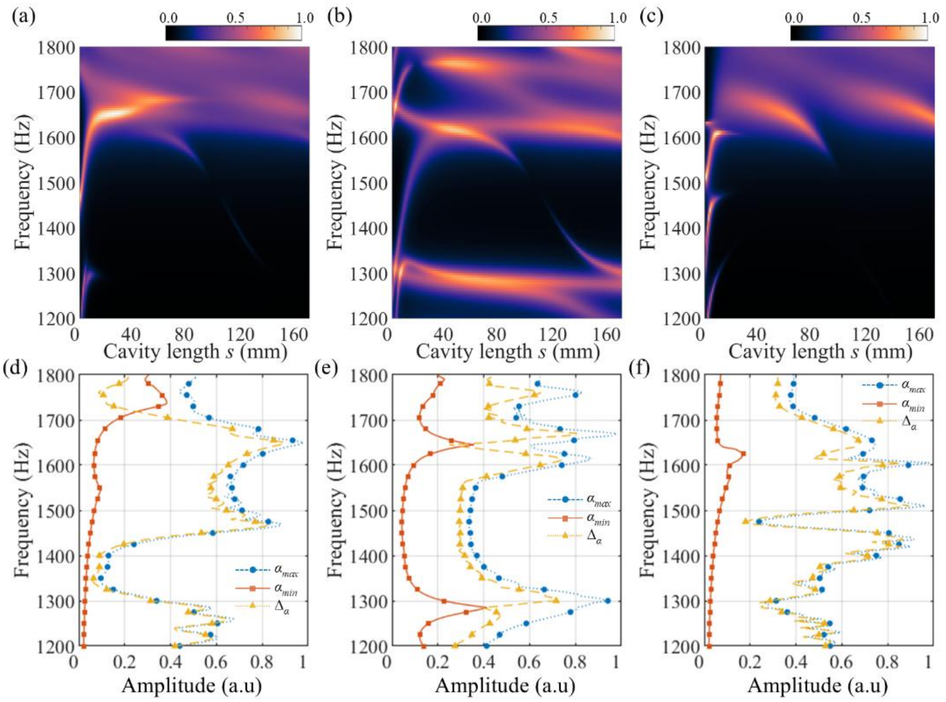

Fig. S31. (a-c) Sound absorption performance over a broad frequency range for three bilayer configurations: (a) (m_1, m_2) = (2, 4), (b) (4, 4), and (c) (2, 3). (d-f) Corresponding tunability of sound absorption for bilayer metasurface configurations of (m_1, m_2) = (d) (2, 4), (e) (4, 4), and (f) (2, 3). Each plot shows the maximum, minimum absorption coefficient as a function of frequency, and the resulting tunability $\Delta\alpha = \alpha_{\text{max}} - \alpha_{\text{min}}$. Note that the results for (m_1, m_2) = (4, 4) are identical those presented in Fig. 5 of the main text.

S.6 Effect of phase gradients on absorption

To clarify how the phase gradient governs the absorption mechanism of the bilayer metasurface, we investigated the absorption characteristics by varying the unit-cell height a. In the main text, a was fixed to 35 mm, and the phase gradient is primarily controlled by varying the number of unit cells m_i required to a 2π phase span within a supercell. For this parameter study, we considered unit-cell heights a = 15 mm, 25 mm, 50 mm, and 75 mm. For each a, we evaluated metasurface layers with m varying from 2 to 6. The resulting supercell period $p(= ma)$ and the corresponding phase gradient ξ are summarized in Table S2. As in the main text, both metasurfaces in the bilayer are assumed to have the same unit-cell height a for each case.

For each a, we computed the absorption coefficient as a function of the cavity spacing s for all bilayer combinations (m_1, m_2). The resulting absorption responses are presented in Fig. S32. Because the absorption behavior varies substantially across configurations, we introduce a compact comparison by categorizing the 25 bilayer combinations (for each a) into five parity groups: PG1 ($m_1 = m_2$), PG2 (both m_1, m_2 odd but $m_1 \neq m_2$), PG3 (m_1 odd, m_2 even), PG4 (both m_1, m_2 even but $m_1 \neq m_2$), and PG5 (m_1 even, m_2 odd). For each group, we evaluated the maximum and average absorption tunability and summarized them in Table S3. Consistent with the main text, bilayer configurations exhibiting high absorption tunability are primarily found in PG3 (odd–even) and PG4 (even–even).

The physical origin of the phase-gradient dependence can be understood in terms of the diffraction component. The transverse wavenumber of the n-th diffracted order is given by $\alpha_n = k_0 \sin\theta_i + \xi n$, where $\xi = 2\pi/(ma)$ is the phase gradient. A diffracted order becomes propagating when $|\alpha_n| < k_0$. When a increases, the phase gradient decreases. Consequently, some of the higher-order components generated by the metasurface fall into the propagating regime. These components contribute to far-field scattering leakage rather than remaining confined as evanescent fields inside the cavity, thereby degrading the absorption performance. In contrast, when a decreases, the associated transverse wavenumbers become larger. As a result, the evanescent orders become more rapidly decaying. This weakens the effective evanescent-wave coupling between the layers, which in turn reduces the absorption enhancement.

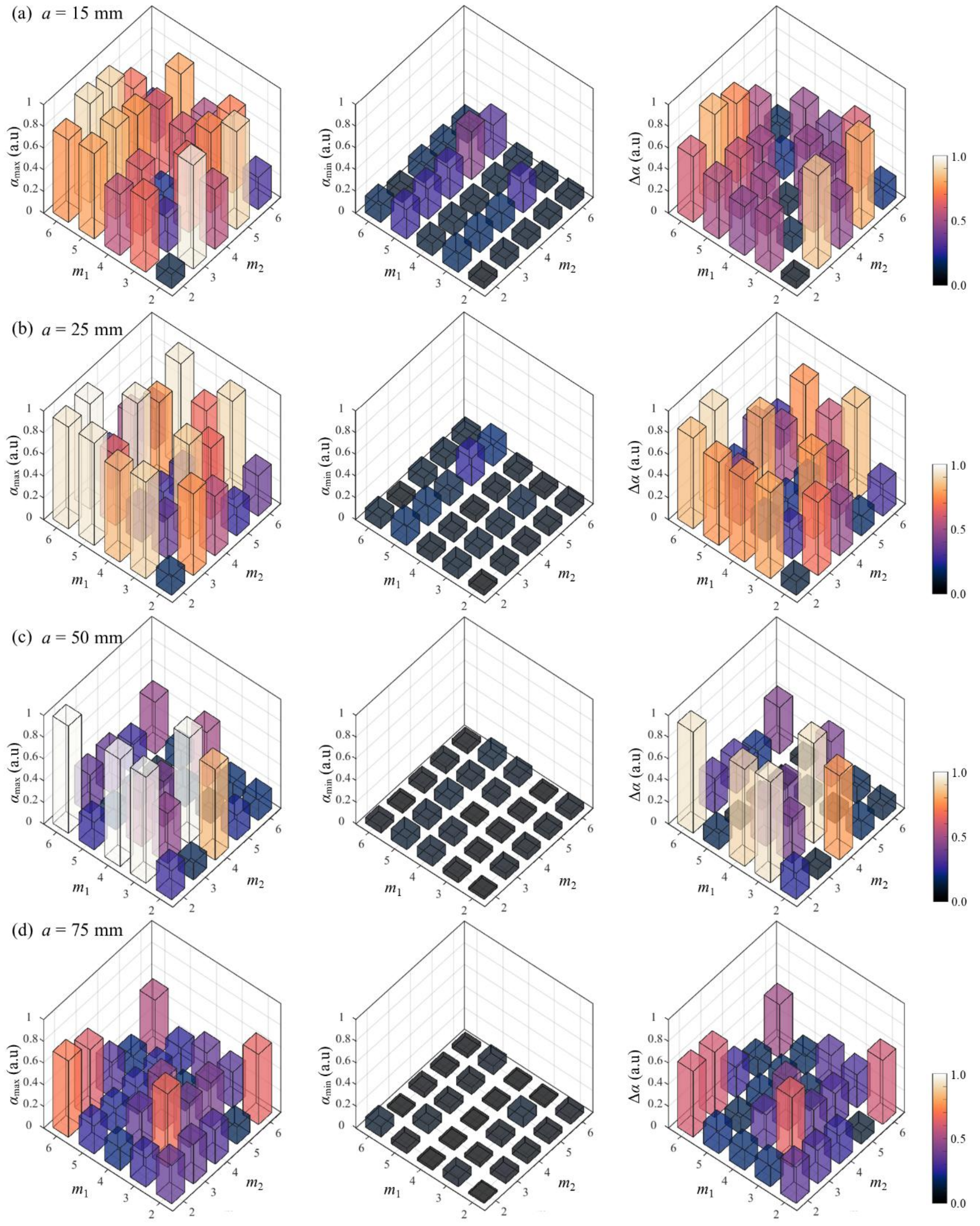

Fig. S32. Tunability of sound absorption for bilayer metasurfaces across different (m_1, m_2) combinations at 1500 Hz: Maximum absorption coefficient, α_{max} (left), minimum absorption coefficient, α_{min} (center), and tunability range (right), defined as $\Delta\alpha = \alpha_{max} - \alpha_{min}$ for unit-cell heights of (a) a = 15 mm, (b) a = 25 mm, (c) a = 50 mm, and (d) a = 75 mm. The corresponding phase gradients are listed in Table S2.

Table S2. Geometric parameters and resulting phase gradient ξ for metasurface layers with m varying from 2 to 6.

		m				
		2	3	4	5	6
a = 15 mm	p	30	45	60	75	90
	ξ	209.4	139.6	104.7	83.8	69.8
a = 25 mm	p	50	75	100	125	150
	ξ	125.7	83.8	62.8	50.3	41.9
a = 35 mm	p	70	105	140	175	210
	ξ	89.8	59.8	44.9	35.9	29.9
a = 50 mm	p	100	150	200	250	300
	ξ	62.8	41.9	31.4	25.1	20.9
a = 75 mm	p	150	225	300	375	450
	ξ	41.9	27.9	20.9	16.8	14.0

Table S3. Summary of absorption performance for bilayer metasurfaces categorized by (m_1, m_2) parity combinations: maximum values of the absorption tunability range, defined as $\Delta_\alpha = \alpha_{max} - \alpha_{min}$, with the corresponding average values provided in parentheses.

	$\Delta_A = \overline{(\alpha_{max} - \alpha_{min})}$					Note
	a = 15 mm	a = 25 mm	a = 35 mm	a = 50 mm	a = 75 mm	
PG1	0.21(0.14)	0.51(0.28)	0.71(0.38)	0.43(0.30)	0.64(0.42)	$m_1 = m_2$
PG2	0.56(0.51)	0.53(0.50)	0.43(0.41)	0.09(0.09)	0.31(0.23)	both m_1, m_2 odd but $m_1 \neq m_2$
PG3	0.61(0.51)	0.84(0.77)	0.95(0.89)	0.93(0.38)	0.34(0.23)	m_1 odd, m_2 even
PG4	0.73(0.48)	0.84(0.53)	0.92(0.61)	0.93(0.58)	0.61(0.37)	both m_1, m_2 even but $m_1 \neq m_2$
PG5	0.86(0.65)	0.90(0.43)	0.65(0.35)	0.41(0.22)	0.60(0.28)	m_1 even, m_2 odd

S.7 Sound absorption performance of arbitrary phase-delay distributions

High sound absorption is generally achieved by suppressing far field radiation while enhancing higher-order evanescent components to confine wave energy and maximize dissipative losses. To verify this, we analyzed the diffraction characteristics of the bilayer metasurface with (m_1, m_2) = (4, 2) configuration, as shown in Fig. S33(a). Figure S33(c) presents the diffraction order components according to absorption performance, showing that the 0th-order reflection component significantly decreases as the absorption coefficient increases while specific higher-order evanescent components (e.g., ±1 and ±3 orders) are enhanced.

To extend our analysis to generalized configurations with arbitrary phase distributions, we examined the diffraction characteristics of a bilayer metasurface with (m_1, m_2) = (4, 4) using 1,000 random phase configurations. As shown in Fig. S33(b), we modeled a supercell comprising a total of 8 unit cells with periodicity in the y-direction without an interlayer cavity, where the phase delay of each unit cell was randomly assigned within a 2π range related to the initial phase offset ϕ_0. The results presented in Fig. S33(d) confirm that high absorption (up to 91%) is strongly correlated with the suppression of the 0th-order mode and the enhancement of evanescent near-field components. Notably, despite the lack of ordered phase gradients, the random phase array with appropriate design leads to the suppression of the far-field radiation channel and the enhancement of the near-field, consistent with the physical behavior observed in the proposed bilayer system.

However, the distinguishing feature of the proposed bilayer system lies in the tunability mechanism. While the arbitrary phase distribution approach requires structural reconfiguration to achieve specific absorption levels (as illustrated in Fig. S33(b)), our approach harnesses the near-field coupling between two fixed metasurfaces as a control parameter. As demonstrated in Fig. S33(a), we achieve a continuous transition from a reflector to a perfect absorber only by adjusting the interlayer spacing s, while keeping the phase-delay distributions invariant.

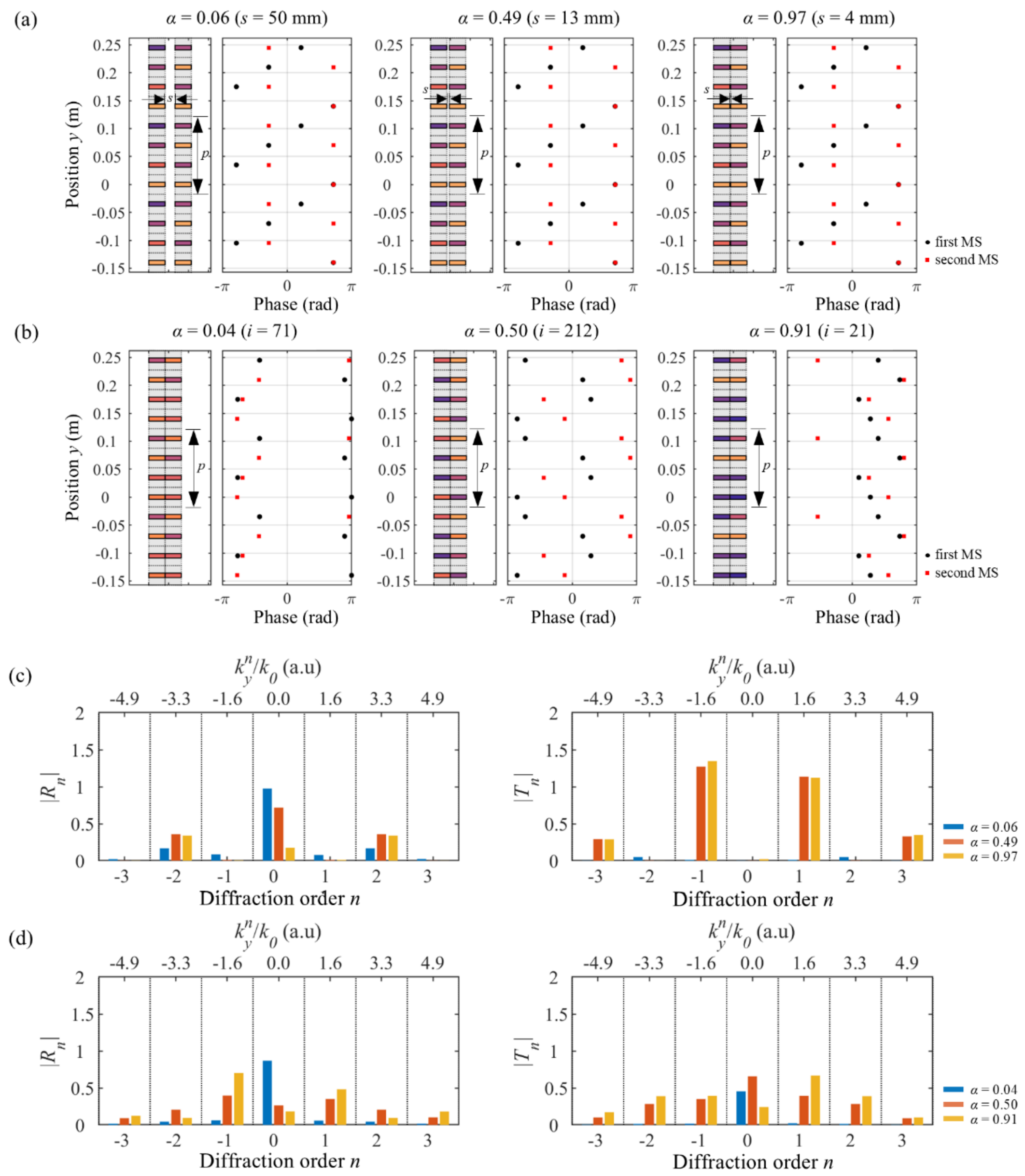

Fig. S33. Phase-delay profiles and corresponding unit cell arrangements for three representative cases exhibiting low, intermediate, and high absorption for (a) the proposed bilayer system and (b) the arbitrary phase design. The black circles and red squares denote the phase delays of the first and second metasurfaces, respectively. Corresponding amplitudes of the reflected ($|R_n|$) and transmitted ($|T_n|$) wave components for various diffraction orders n for (c) the proposed bilayer system and (d) the arbitrary phase design.
